# Factors driving the global decline of cycad diversity

**DOI:** 10.1093/aobpla/plx022

**Published:** 2017-05-29

**Authors:** Ledile T. Mankga, Kowiyou Yessoufou

**Affiliations:** 1 Department of Life and Consumer Sciences, University of South Africa, Florida campus, Florida 1710, South Africa.; 2 Department of Geography, Environmental Management and Energy Studies, University of Johannesburg, APK Campus, Auckland Park 2006, Johannesburg, South Africa.

**Keywords:** Anthropogenic pressure, cycad ecology and biology, data deficient species, evolutionary distinctiveness, species loss, tree of life

## Abstract

Mounting evidence indicates that we are witnessing the sixth mass extinction period. Given the important goods and services biodiversity delivers to humans, there is a need for a continued commitment to investigate what pre-disposes some taxa to greater risk of extinction. Here, we investigate this question using a phylogenetic comparative method and fitting a cumulative link mixed effect model on biological, ecological and evolutionary data of cycads, the most threatened lineage in the plant kingdom. We identified nine groups of threats to cycads, with habitat loss, over-collection, fire and reproduction failure being the most prominent, but only four of these threats (habitat loss, over-collection, medicinal uses and reproduction failure) clustered on the cycad tree of life. This clustering suggests that closely related species may be exposed to similar threats, perhaps because of geographic regionalization of cycad genera. Nonetheless, the diversity of threats and several variables linked to the biology and ecology of cycads correlate with extinction risk (e.g. altitude, height, diameter, geographic range), and different variables seem to be linked to different IUCN status of cycads. Although their predictive power is generally < 50 %, geographic range and maximum diameter stood out as the best predictors particularly for the Vulnerable (VU) category, with a predictive power of 87 % and 69 %, respectively. Using our best model for VU, we predicted all five Data Deficient (DD) species of cycads to be in the VU category. Collectively, our results elucidate the pattern of extinction risk in cycads and, since most threats that we identified as drivers of extinction risk of cycads are anthropogenically mediated, we recommend stronger legislation to regulate human–cycad interactions and the commitment of all governments globally to implement this regulation.

## Teaser

We have entered the sixth mass extinction period where species diversity is being lost at an unprecedented rate. Specifically, cycads are the most threatened plant group in the plant kingdom, and the present study investigated the factors that correlate with the extinction risk of cycads. We identified nine groups of threats; some of them are not randomly distributed along the cycad tree of life, suggesting that closely related species may be exposed to similar threats. We also identified a number of significant predictors of extinction risk linked to the biology and ecology of cycads. Collectively, our results elucidate the pattern of extinction risk in cycads.

## Introduction

A better understanding of the drivers of extinction risk is necessary to inform conservation decisions, and predicting future risk could be informed of the historical extinction events. However, there is a disparity in efforts devoted to unravelling these drivers and the extinction risk patterns of vertebrates (e.g. Cooper *et al.* 2008; Davies and Yessoufou 2013; Luiz *et al.* 2016; Schachat *et al.* 2016; Veron *et al.* 2016; see further references in Pellens and Grandcolas 2016) in comparison to plants, and the only few extinction risk studies that focus on plants prioritize angiosperms (e.g. Sodhi *et al.* 2008; Yessoufou *et al.* 2012; Daru *et al.* 2013; Leao *et al.* 2014). As a result, we are comparatively well-informed of the pre-disposition of vertebrates and, to a lesser extent, angiosperms, to extinction risk as well as how their phylogenetic trees would be affected by species loss (Davies *et al.* 2011; Mooers *et al.* 2012; Davies and Yessoufou 2013). In contrast, such knowledge is yet to be well-demonstrated for gymnosperms, although the latter group is more threatened than angiosperm (e.g. ∼70 % of cycads are threatened, IUCN 2010; Yessoufou *et al.* 2017).

Cycads are a group of gymnosperm of particular interest due to their evolutionary history (Nagalingum *et al.* 2011; Yessoufou *et al.* 2014; Condamine *et al.* 2015) and their morphological features shared between ferns and angiosperms (Norstog and Nicholls, 1997; Brenner *et al.* 2003). Their origin dated back to ∼300 million years ago (Hendricks 1987), and in the Mesozoic era, cycads exhibited a worldwide distribution (Hermsen *et al.* 2009). However, the age of the extant cycads is much younger (12–2 Ma, Nagalingum *et al.* 2011), and they are restricted to tropical and subtropical regions of the world. Unfortunately, 70 % of all the 339 cycad taxa (Yessoufou *et al.* 2017) are threatened with high risk of extinction (IUCN, 2010; Osborne *et al.* 2012; Yessoufou *et al.* 2017). Current knowledge indicates that ecological or biological factors (Sodhi *et al.* 2008; Yessoufou *et al.* 2012) as well as evolutionary history (Davies *et al.* 2011) pre-dispose a particular taxonomic group to risk of extinction. For example, life history trait such as body size pre-dispose vertebrates to extinction risk (Cardillo 2003), but the role of size in pre-disposing plants to extinction remains debatable (Freville *et al.* 2007; Bradshaw *et al.* 2008; Sodhi *et al.* 2008). In contrast, evidence suggests that extinction risk in plants may be rather linked to their evolutionary rather than life history (Lozano and Schawartz 2005; Vamosi and Wilson 2008; Davies *et al.* 2011; Daru *et al.* 2013).

On the basis of this knowledge, we compiled a list of putative biological and ecological parameters linked to extinction risk in previous studies. This includes altitude, diameter, diversity of threats (i.e. number of threats recorded for each species), generation time, geographic range and height. For example, high-altitude habitats are usually considered a ‘safe heaven’ for ancient but threatened taxa (Fjeldsa and Lovett 1997; Fjeldsa et al. 2012). Also, an early study found a higher richness of threatened species at high altitude (Yessoufou *et al.* 2012). In addition, extinction risk in animals has also been linked to body size, generation time and geographic range with the expectations that species with larger size, longer generation time and smaller geographic range would be more at risk (Bennett and Owens 1997; Russell *et al.* 1998; Purvis *et al.* 2000; Cardillo 2003; Fisher and Owens 2004; Cooper *et al.* 2008, IUCN 2010). The representatives of body size in the present study are diameter and height (see also Sodhi *et al.* 2008).

Furthermore, evidence that evolutionarily younger or older taxa tend to me more at risk is indicative of an evolutionary pre-disposition to extinction (Purvis *et al.* 2000; Vamosi and Wilson 2008; Davies *et al.* 2011; Daru *et al.* 2013). Also, because phylogenetically conserved traits can be linked to extinction (e.g. phenology, Willis *et al.* 2008), it becomes necessary to assess the distribution of extinction risk along a phylogenetic tree (Fritz and Purvis 2010; Davies *et al.* 2011; Yessoufou *et al.* 2012), although extinction risk is not an evolving trait (Grandcolas *et al.* 2011). Such a phylogenetic signal analysis of extinction risk would help predict whether unrelated species (e.g. species with high value of evolutionary distinctiveness (ED); Isaac *et al.* 2007) or closely related ones are more at risk (Purvis *et al.* 2000). However, the phylogenetic analysis of extinction risk is traditionally conducted based on IUCN threat categories (Davies *et al.* 2011). This tradition is recently showed to be potentially misleading as long as the drivers of extinction risk are not taken into consideration (see Schachat *et al.* 2016). Similarly, conservation decisions could be further misled if the threat status of some species remains unknown (e.g. Data Deficient, DD, species; Luiz *et al.* 2016; Veron *et al.* 2016).

In the present study, our objective is to provide a better explanation of extinction risk in the cycad group. Specifically, we identified and categorized all threats to cycads, tested for phylogenetic signal in the threat categories, and generate the best model of extinction risk that was then used to predict the threat status of DD species.

## Methods

### Global cycad diversity, IUCN status and categories of threats

In a recent study, our research group compiled a list of 339 cycad taxa following a thorough literature search (e.g. Lindström 2009; Nagalingum *et al.* 2011; Osborne *et al.* 2012) and taking into account some synonymous names **[see Supporting Information I]**. In the same study, IUCN threat categories for all taxa were also compiled (www.redlist.org, August 2016; Osborne *et al.* 2012): DD (five taxa), Least Concern (LC: 47 taxa), Near Threatened (NT: 68 taxa), Vulnerable (VU: 78 taxa), Endangered (EN: 70) and Critically endangered (CR: 67 taxa). In the present study, we complemented these data with additional information on different threats to cycads available from various sources including the IUCN database (www.redlist.org, August 2016) **[seeSupporting Information—Table S1]**.

### Cycad tree of life

The phylogenetic tree used in this study is the complete cycad tree comprising 339 taxa recently assembled in our research group (see Yessoufou *et al.* 2017) by combining DNA sequences of the nuclear region PHYP for 199 species (Nagalingum *et al.* 2011) and taxonomic information following the Thomas *et al.*’s (2013) approach. This tree is submitted to TreeBase with the submission ID # 20161 and the details of tree reconstruction are available in a recent paper that we published (Yessoufou *et al.* 2017).

### Potential predictors of IUCN status

To fit predictive models of IUCN status for all cycads, we compiled a list of putative variables including altitude, diameter, diversity of threats, ED, generation time, geographic range and height. We recorded the minimum and maximum of the altitudinal occurrence of each species from IUCN (2010). Two types of diameters were recorded, the minimum and the maximum diameter. The diversity of threats was defined as the number of threat categories (as defined above) recorded for each species. ED is a metric that approximates the evolutionary ages of each species such that a species with a higher ED value is subtended on a phylogeny by a longer branch (Isaac *et al.* 2007). ED values for all cycads were compiled from Yessoufou *et al.* (2017) **[see Supporting Information I]**. Data on generation time were retrieved from the IUCN database (www.redlist.org, August 2016). Geographic range data were compiled in two ways; first as surface area of geographic ranges (in km^2^) and these data were retrieved from IUCN (2010) and, second, as the number of locations where a species occurs (defined in Osborne *et al.* 2012). Finally, we documented the minimum and maximum height for each cycad species also from IUCN. Overall, 11 variables were included in our predictive models, and values for all these variables are presented in **[Supporting Information—Table S1]**.

### Data analysis

All analyses were conducted in R (R Core Team 2015) and detailed below. Prior to analyses we checked for correlations among all the 11 variables to avoid redundancy. We found that minimum altitude and maximum altitude do correlate, as well as minimum and maximum height **[see Supporting Information—Fig. S1]**. We therefore discarded maximum altitude and maximum height, implying that our analysis on modelling presented below focused only on the remaining nine variables.

### Phylogenetic signal in threat groups to cycads

Each threat identified was coded as follows; 1: when a threat is reported for a species; 0: when a threat is not reported for a species and NA: when information for a species were missing for a given threat **[seeSupporting Information—Table S1]**. Prior to any analysis, missing data were explored using a combination of graphical displays (Prantner 2011), first for threats **[see Supporting Information—Fig. S2]**, then for predictors of IUCN status **[see Supporting Information—Figs S3 and S4]**, and last, we used the *k*-nearest-neighbour imputation method implemented in the R package VIM (Templ *et al.* 2016) to impute NA values. Next, we applied the *D* statistic (Fritz and Purvis 2010) to assess the phylogenetic signal in each threat using the complete cycad phylogeny of Yessoufou *et al.* (2017). The *D* statistic provides an estimate of phylogenetic conservatism for binary traits that can be compared with both a random shuffle of trait values at the tips of a phylogeny and a Brownian threshold model (BM; Fritz and Purvis 2010), but we report here only the significance at random. When *D* = 1 then traits are randomly distributed at the tips of the phylogeny; *D* = 0 corresponds to a BM model; *D* < 0 signifies traits are highly conserved, whereas *D* > 1 signifies traits are over-dispersed on phylogenetic tree. If a *D* value falls between 0 and 1, we tested whether this value is statistically different from 1 (random); if so, then we concluded that the observed *D* value is non-random. If *D* value is not statistically different from 1, then the observed value is considered as random.

### Predictive models for extinction risk of cycads

We explored the power of each of our nine variables to predict the IUCN threat status of cycad species by fitting the cumulative link mixed effect model (CLMM; Christensen 2013). The IUCN status is a ranked categorical status defined as LC *< *NT *< *VU *< *EN *< *CR. We preferred the CLMM approach as our modelling method to the machine-learning methods based on a number of advantages the CLMM provides (see Luiz *et al.* 2016 for details). In summary, CLMM is a better approach as it allows a direct analysis of ranked categorical variables (here IUCN categories: LC < NT < VU < EN < CR) as response variables without necessarily converting them into numerical values (and such conversion is the tradition; e.g. see Mooers *et al.* 2008; Davies *et al.* 2011 or Yessoufou *et al.* 2012). In so doing, CLMM has the advantage of preserving the variance structure of the original ordinal ranks of the categorical response variables, and thus prevents the loss of information generally observed when categorical values are either converted into numerical values or grouped into binomial classifications (e.g. non-threatened vs. threatened categories). CLMM also prevents an unnecessary elevated type I error generally observed when IUCN categories are converted into numerical values where differences between adjacent risk levels are assumed equivalent (e.g. LC = 0 and NT = 1 or EN = 3 and CR = 5).

In our models, the response variable is the IUCN status, and the dependent variables used as fixed effects are altitude, diameter, diversity of threats, ED, generation time, geographic range and height. We also included the taxonomic ranks ‘Genus’ within ‘Family’ and ‘Family’ within ‘Order’ as a random effect in our models to account for potential effects of shared ancestry, using the R function ‘clmm’ (package ‘ordinal’ Christensen 2013).

Two types of models were generated, univariate and multivariate. For our multivariate model, we conducted model selection using a backward stepwise removal of non-significant fixed-effect terms from the full model, based on log-likelihood ratio tests. The predictive power of our model fit was quantified as the percentage of species whose IUCN status are correctly predicted by the model; this is referred to, in Luiz *et al.* (2016), as Percentage Correct Classified (PCC). The PCC value was calculated in two ways; first, PCC was calculated for each best model identified considering all species (i.e. overall predictive power of each model) and second, PCC was also calculated for each best model considering species in each IUCN category (i.e. predictive power of each model per IUCN category). Models were fitted using the function ‘clm’ from the R package ‘ordinal’ (Christensen 2013). We used the coefficients of our final model to estimate the IUCN threat status of the five DD species (the R function ‘predict’ implemented in the package ‘ordinal’).

## Results

**Figure 1.**
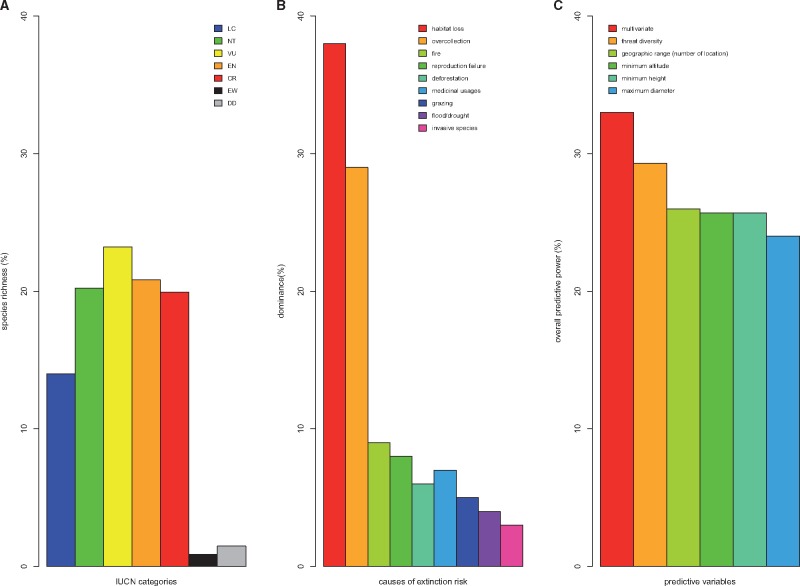
General pattern of extinction risk in the cycad group. (A) Cycad species richness in each IUCN threat categories (LC, least concern; NT, near threatened; VU, vulnerable; EN, endangered; CR, critically endangered; DD, data deficient; EW, extinct in the wild); (B) identified causes of threats to cycad globally; (C) the overall predictive power of all significant correlates of extinction risk of cycads. Multivariate = the best multivariate model; this model includes maximum diameter, geographic range (measured as number of locations of species occurrence) and diversity of threats (Table 1).

**Figure 2.**
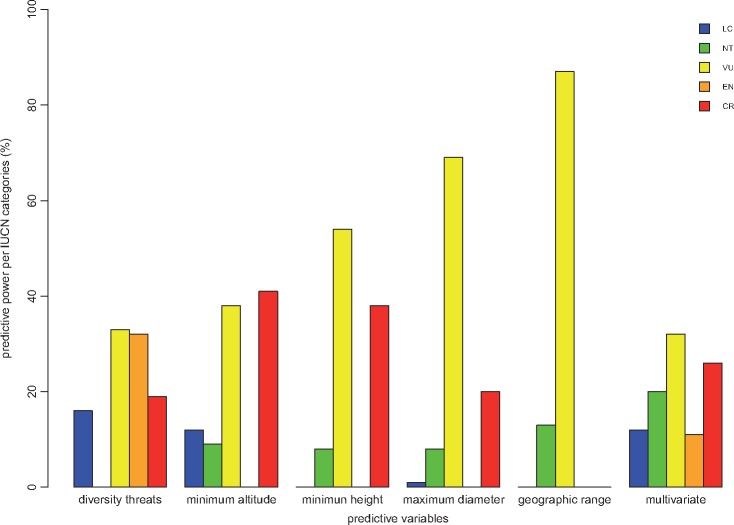
Predictive powers of all significant correlates of extinction risk in each IUCN categories. LC, least concern; NT, near threatened; VU, vulnerable; EN, endangered; CR, critically endangered.

Cycad group comprises ∼64 % of threatened species in the categories VU (23 %), EN (21 %) and CR (20 %), and almost 1 % and 2 % of species are in the categories Extinct in the Wild and DD, respectively (Fig. 1A). Such a high level of extinction risk is driven mainly by nine categories of threats including pre-dominantly habitat loss (38 %), over-collection (29 %), fire (9 %) and reproduction failure (8 %) and to a lesser extent invasive species (3 %; Fig. 1B). In total, we identified five variables that correlate significantly with the extinction risk, of which three correlate negatively (geographic range measured as number of locations of species occurrence), minimum height and maximum diameter) and the remaining two correlate positively (threat diversity, i.e. number of threats facing each species and minimum altitude; Table 1, Fig. 1C). However, our multivariate model (that includes threat diversity, maximum diameter and geographic range) has the overall highest predictive power of extinction risk (PCC = 33 %) and maximum diameter the lowest (PCC = 24 %; Fig. 1C). At IUCN category level, although we found that all models (uni- and multivariate) yielded their best prediction for VU category (except for minimum altitude, Fig. 2), we also found that different variables are good predictors of different IUCN categories. For example, geographic range (measured as number of locations), maximum diameter and minimum height are excellent predictors of VU (87 %, 69 % and 54 %, respectively); minimum altitude for CR (41 %), diversity of threats for EN (32 %) and VU (33 %; Fig. 2).

**Table 1.** Parameters of the cumulative linear mixed effects models with the IUCN red list category as an ordinal categorical response variable. Significant variables are indicated by stars (*) and the number of stars indicate the level of significance. NS, not significant.

	Variables	Estimate	Standard error	*Z* values	Probability values
Univariate model	Diversity of threats (log +1)	1.531	0.244	6.260	*P* < 0.001***
	ED (log)	0.500	0.256	1.953	*P* = 0.05
	Range (km^2^) (log)	−0.087	0.055	−1.572	*P* = 0.116
	Minimum altitude (log alt + 1)	0.234	0.061	3.83	*P* < 0.001***
	Minimum height (log)	−0.287	0.111	−2.594	*P* = 0.009**
	Generation time (log)	−0.039	0.312	−0.127	*P* = 0.899^NS^
	Maximum diameter (log)	−0404	0.185	−2.177	*P* = 0.029*
	Geographic range (number of locations) (log)	−0.625	0.154	−4.036	*P* < 0.001***
Multivariate model	Maximum diameter (log)	−0.471	0.189	−2.491	*P* = 0.012*
	Geographic range (log)	−0.647	0.156	−4.139	*P* < 0.001***
	Diversity of threats (log)	1.554	0.245	6.319	*P* < 0.001***

All these models indicate that the DD species are threatened (VU, EN or CR). In particular, based on the geographic range (number of locations) that showed the highest predictive power for VU (87 %), all DD species (*Cycas aenigma*, *C. indica*, *C. sphaerica*, *Ceratozamia brevifrons* and *Zamia lindleyi*) are predicted to be in the VU category.

**Figure 3.**
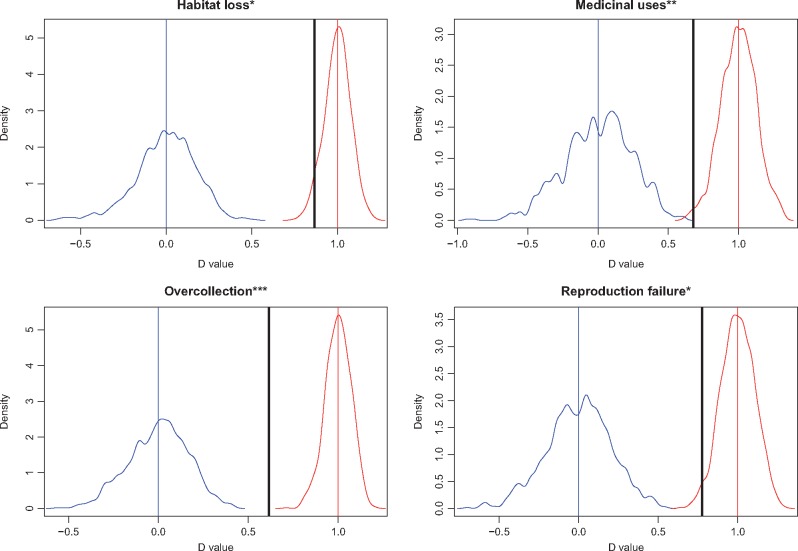
Results of the tests of phylogenetic signal in the causes of extinction risk using Fritz and Purvis’ (2010) *D* statistic. The graph in blue is the distribution of *D* values assuming a Brownian Motion (BM) model, and the blue vertical line indicates *D* = 0 (when the phylogenetic distribution of a parameter is no different from BM). The graph in red is the distribution of *D* values assuming a random model, and the red vertical line indicates *D* = 1 (when the phylogenetic distribution of a parameter is no different from random). The bold black vertical lines indicate the observed *D* values. The number of * is indicative of the significance level of the observed *D* values.

Finally, we further explored the phylogenetic pre-disposition of cycads to extinction risk. Of the nine categories of threats that we identified, we found evidence of phylogenetic signal in only four: habitat loss, medicinal uses, over-collection and reproduction failure (Fig. 3).

## Discussion

We summarized all threats to cycads into nine categories, of which seven are directly linked to human activities (habitat loss, over-collection, fire, deforestation, medicinal usages, grazing, invasive alien plants), one to the biology of cycad (reproduction failure) and the last one is linked to climate change impacts (flood/drought). This is indicative of the prominent role human plays in driving the loss of biodiversity (Wake and Vredenburg 2008), particularly under the tropic (cycads are mainly tropical), thus supporting the well-known ‘tropical biodiversity crisis’ (Vamosi and Vamosi 2008).

Although there is a general trend for closely related species to be threatened (phylogenetic signal) irrespective of the taxonomic group at hand (Purvis, 2008; Purvis *et al.* 2000; Davies *et al.* 2011; Yessoufou *et al.* 2012; see Yessoufou and Davies 2016 for further references), a recent study revealed that such evidence does not hold for cycads, i.e. threatened cycad species are not significantly clustered on the cycad tree of life (Yessoufou *et al.* 2017). The phylogenetic signal analysis is traditionally explored on IUCN threat categories (e.g. Fritz and Purvis 2010; Davies *et al.* 2011; Yessoufou *et al.* 2017), but a recent study demonstrated convincingly that the causes of extinction rather than the extinction risk status should be integrated into the phylogenetic comparative analysis of extinction risk (Schachat *et al.* 2016). As opposed to Yessoufou *et al.* (2017) who found no evidence for a phylogenetically patterned extinction risk for cycads, our results here indicate that certain causes of extinction of cycad species can be linked to phylogenetic pre-disposition. For example, we found evidence that phylogenetically closely related species are more threatened than expected by habitat loss, over-collection, medicinal uses and reproduction failure. This finding suggests a phylogenetic pre-disposition of cycads to extinction such that closely related species may share similar vulnerabilities in the face of similar threats. Such phylogenetic pre-disposition could be the result of closely related species sharing similar life history traits that evolve along the phylogeny. It could also be because closely related species are in fact exposed to similar threats, given the geographic regionalization of cycad genera (e.g. all species within the genus *Encephalartos* occur in Africa, and all species in the genus *Ceratozamia* occur in the New World, etc.).

We further tested for correlates of extinction risk of cycads fitting a CLMM on nine biological, ecological and evolutionary variables. Two of these variables correlate positively with extinction risk (diversity of threats and minimum altitude). Our finding that species facing a high diversity (number) of threat are more at risk of extinction is not a surprise. So is the positive correlation of extinction with altitude, as higher altitude may be playing the role of refugia for species that are threatened at lower altitude due to human pressure (Sandel *et al.* 2011; Yessoufou *et al.* 2012; White and Bennett 2015). Positive correlation of ED, a phylogenetic metric, with extinction risk was previously reported for cycads (e.g. Yessoufou *et al.* 2017), and this provides support for a phylogenetic pre-disposition of plants to extinction (Vamosi and Wilson 2008; Davies *et al.* 2011; Condamine *et al.* 2013; Daru *et al.* 2013). How does phylogenetic history pre-dispose plant to extinction risk remains to be elucidated. In the Cape Floristic Region, young plant lineages are more at risk (Davies *et al.* 2011) whilst the opposite trend was recently reported for plant lineages in mangrove ecosystems (Daru *et al.* 2013). However, the correlation between ED and extinction risk reported in Yessoufou *et al.* (2017) is not confirmed in the present study for cycads, and this is because Yessoufou *et al.* (2017) did not account for shared ancestry among species in their analysis.

Furthermore, geographic range (measured as number of geographic locations where species are found; Osborne *et al.* 2012), minimum height and maximum diameter show a negative correlation with extinction risk. This result for geographic range is not surprising as this variable is one of the key of IUCN threat categorization system (IUCN 2010). However, a negative correlation of height with extinction is counterintuitive, as we would expect taller species to be more at risk because they are easy to spot by illegal cycad collectors. Our finding could perhaps be an indication that shorter cycads might be more VU to a number of threats that we identified above, including flood, invasive plants, and particularly to grazing and fire (see lanky and corky strategy of Dantas and Pausas 2013). The correlation of height with extinction risk could also result in the correlation that we found for diameter, given the well-known allometric relationship between height and diameter (e.g. Mugasha *et al.* 2013). In particular, species with small diameter may have invested more in vertical growth (i.e. height), and in light of the negative correlation between height and extinction risk, the negative correlation between diameter and extinction risk becomes meaningful.

Despite these significant correlations, the overall predictive power of the models generated are only between 24 % and 33 %, indicating that many other variables driving the extinction risk of cycads are not included in our study. This provides room for further investigations of the pre-disposition of cycad to high risk of extinction. In contrast, we found a strong predictive power while looking at each IUCN threat category level particularly for geographic range (number of locations of species occurrence), which predicts correctly 87 % of VU status. Such strong predictive power per IUCN threat category was used to clarify the status of DD species. All five DD cycad species were predicted to be in the VU category, thus adding five more species to the threatened cycad species richness.

## Conclusion

In the present study, we explore what pre-disposes cycads to high risk of extinction. Mainly human induced pressures (e.g. habitat loss, grazing, fire, medicinal usages, etc.), the biology of cycads (e.g. reproduction failure) and climate related variables (e.g. drought, flood) threaten cycad diversity, putting at risk the evolutionary history accumulated in the cycad tree of life over million years. We acknowledge that many other threats particularly linked to the biology and ecology of cycad (e.g. dispersal ability, availability of pollinators, cycad–pollinator interactions, etc.) could also be playing a role in shaping the current extinction risk pattern, but these variables are not evaluated in the present study. We explicitly explore this pattern and reveal a phylogenetic basis for extinction risk of cycad such that phylogenetically closely related species are exposed to similar threats. It is well-established that excluding DD species from extinction risk analysis (this is the tradition, Bielby *et al.* 2006; Yessoufou *et al.* 2012) is likely to induce bias in decision-making process (Whittaker *et al.* 2005; Luiz *et al.* 2016) and could therefore mislead our understanding of how extinction risk may prune the tree of life (Veron *et al.* 2016). More critically, we identified significant (statistically) correlates of extinction risk for cycads, and used predictive models to determine the IUCN threat status of the five DD cycad species. As such, our study allows for a comprehensive picture of the extinction pattern in cycad group and elucidates the evolutionary pre-disposition of plants to extinction risk.

## Sources of Funding

Funding for this study was provided to Dr Kowiyou Yessoufou through the South Africa’s National Research Foundation Grant No: 103944, South Africa, and the 2016 Research Prize of the Société Botanique de France, France (No grant number).

## Contributions by the Authors

K.Y. designed the project, L.T.M. and K.Y. collected and analysed the data, L.T.M. and K.Y. wrote the manuscript and K.Y. coordinated the whole project.

## Conflict of Interest Statement

None declared.

## Supplementary Material

Supplementary Data
