# Supplementary material for: Factors driving the global decline of cycad diversity
Source: AoB Plants. 2017 May 29;9(4):plx022. doi: 10.1093/aobpla/plx022 (PMC5506724; doi:10.1093/aobpla/plx022)
Supplement: Supplementary Data [file plx022_supp.docx]

**Factors driving the global decline of cycad diversity**

**Table S1** Raw data for all ecological, biological and evolutionary information used in this study

| Species | Global_Endangerment | Habitat_loss_Destruction | Deforestation | Medicinal_uses | Overcollection_Poaching | Flood_Drought | Reproduction_failure | Grazing | Invasive_plants | Fire | No_threats | ED | range_km2 | altitude_min | altitude_max | threat_status | Height_min | Height_max | Generation_time | Diameter_min | Diameter_max | Geographic_range |
| --- | --- | --- | --- | --- | --- | --- | --- | --- | --- | --- | --- | --- | --- | --- | --- | --- | --- | --- | --- | --- | --- | --- |
| Bowenia_serrulata | LC | 1 | 0 | 0 | 0 | 0 | 0 | 0 | 0 | 0 | 1 | 50.14995 | NA | 30 | 150 | non-threatened | NA | NA | 30 | 0.3 | 0.25 | 1 |
| Bowenia_spectabilis | LC | 1 | 0 | 0 | 0 | 0 | 0 | 0 | 0 | 0 | 1 | 50.14995 | NA | 0 | 750 | non-threatened | NA | NA | 30 | NA | 10 | 1 |
| Ceratozamia_alvarezii | EN | 1 | 0 | 0 | 1 | 0 | 0 | 0 | 0 | 0 | 2 | 39.5459 | 16 | NA | NA | threatened | 0.1 | 0.5 | 45 | 0.089 | 0.175 | 1 |
| Ceratozamia_becerrae | EN | 1 | 0 | 0 | 0 | 0 | 0 | 0 | 0 | 1 | 2 | 30.23118 | 1000 | NA | NA | threatened | 0.1034 | 0.31 | 45 | 0.0548 | 0.1047 | 2 |
| Ceratozamia_chimalapensis | CR | 1 | 0 | 0 | 0 | 0 | 0 | 0 | 0 | 1 | 2 | 39.5459 | NA | NA | NA | threatened | 0.2 | 0.1 | NA | 0.178 | 0.331 | 1 |
| Ceratozamia_decumbens | CR | 1 | 0 | 0 | 1 | 0 | 0 | 0 | 0 | 0 | 2 | 33.73552 | NA | NA | NA | threatened | 0.09 | 0.2 | NA | 0.08 | 14 | 1 |
| Ceratozamia_euryphyllidia | CR | 1 | 0 | 0 | 0 | 0 | 0 | 0 | 0 | 0 | 1 | 20.08057 | NA | NA | NA | threatened | 0.1034 | 0.31 | 45 | 0.548 | 0.1047 | 2 |
| Ceratozamia_fuscoviridis | CR | 1 | 1 | 1 | 0 | 0 | 0 | 0 | 0 | 0 | 3 | 39.03437 | NA | NA | NA | threatened | 0.165 | 0.36 | NA | 0.14 | 0.22 | 1 |
| Ceratozamia_hildae | EN | 1 | 0 | 0 | 1 | 0 | 0 | 0 | 0 | 0 | 2 | 36.39392 | NA | NA | NA | threatened | 0.1 | 0.2 | 45 | 0.05 | 0.25 | 3 |
| Ceratozamia_hondurensis | CR | 1 | 1 | 0 | 1 | 1 | 0 | 1 | 0 | 0 | 5 | 19.85266 | NA | NA | NA | threatened | NA | NA | 45 | NA | NA | 1 |
| Ceratozamia_huastecorum | CR | NA | NA | NA | NA | NA | NA | NA | NA | NA | 0 | 39.03437 | NA | NA | NA | threatened | NA | NA | 45 | NA | NA | 1 |
| Ceratozamia_kuesteriana | CR | 1 | 0 | 0 | 1 | 0 | 0 | 0 | 0 | 0 | 2 | 25.5329 | NA | NA | NA | threatened | NA | NA | 45 | NA | NA | 1 |
| Ceratozamia_latifolia | EN | 1 | 0 | 0 | 1 | 0 | 0 | 0 | 0 | 0 | 2 | 20.70881 | NA | NA | NA | threatened | 0.1 | 0.2 | 45 | NA | 0.1 | 4 |
| Ceratozamia_matudae | EN | 1 | 0 | 0 | 0 | 0 | 1 | 0 | 0 | 0 | 2 | 24.96245 | 5000 | NA | NA | threatened | 0.1 | 0.5 | 45 | 0.89 | 0.175 | 3 |
| Ceratozamia_mexicana | VU | 1 | 1 | 0 | 1 | 0 | 0 | 0 | 0 | 0 | 3 | 20.08057 | NA | NA | NA | threatened | NA | 1 | 45 | 0.08 | 0.2 | 2 |
| Ceratozamia_microstrobila | VU | 1 | 0 | 0 | 0 | 0 | 0 | 0 | 0 | 0 | 1 | 41.76929 | 1000 | NA | NA | threatened | NA | 0.25 | 45 | NA | 10 | 2 |
| Ceratozamia_miqueliana | CR | 1 | 0 | 0 | 1 | 0 | 0 | 0 | 0 | 0 | 2 | 22.91372 | NA | NA | NA | threatened | 0.1034 | 0.31 | 45 | 0.548 | 0.1047 | 3 |
| Ceratozamia_mirandae | EN | 1 | 0 | 0 | 1 | 0 | 0 | 0 | 0 | 1 | 3 | 34.77023 | NA | NA | NA | threatened | NA | NA | 45 | NA | NA | 1 |
| Ceratozamia_mixeorum | EN | 1 | 1 | 0 | 0 | 0 | 0 | 0 | 0 | 0 | 2 | 42.82403 | 25 | NA | NA | threatened | 0.34 | 1.25 | 45 | 0.14 | 0.18 | 1 |
| Ceratozamia_morettii | EN | 0 | 0 | 0 | 1 | 0 | 0 | 0 | 0 | 0 | 1 | 47.26377 | 10 | NA | NA | threatened | NA | 0.3 | 45 | NA | 0.08 | 1 |
| Ceratozamia_norstogii | EN | 0 | 0 | 0 | 1 | 0 | 0 | 0 | 0 | 0 | 1 | 22.39375 | 1100 | NA | NA | threatened | 0.1 | 0.5 | 45 | 0.089 | 0.175 | 2 |
| Ceratozamia_robusta | EN | 1 | 0 | 0 | 1 | 0 | 0 | 0 | 0 | 1 | 3 | 20.70881 | NA | NA | NA | threatened | 1.5 | 2 | 45 | NA | 0.3 | 11 |
| Ceratozamia_sabatoi | EN | 1 | 0 | 0 | 0 | 0 | 0 | 1 | 0 | 0 | 2 | 25.5329 | NA | NA | NA | threatened | 0.1 | 0.5 | 45 | 0.089 | 0.175 | 2 |
| Ceratozamia_santillanii | CR | NA | NA | NA | NA | NA | NA | NA | NA | NA | 0 | 33.73552 | NA | NA | NA | threatened | NA | NA | 45 | NA | NA | 1 |
| Ceratozamia_vovidesii | VU | 1 | 0 | 0 | 0 | 0 | 0 | 0 | 0 | 1 | 2 | 25.92663 | NA | 1000 | 1700 | threatened | NA | NA | 45 | NA | NA | 1 |
| Ceratozamia_whitelockiana | EN | 1 | 0 | 0 | 0 | 0 | 0 | 0 | 0 | 1 | 2 | 19.85266 | NA | NA | NA | threatened | 0.2 | 0.3 | 45 | 0.12 | 0.18 | 1 |
| Ceratozamia_zaragozae | CR | 0 | 0 | 0 | 1 | 0 | 0 | 0 | 0 | 0 | 1 | 30.23118 | 45 | NA | NA | threatened | NA | NA | 45 | NA | NA | 1 |
| Ceratozamia_zoquorum | CR | 1 | 0 | 0 | 1 | 0 | 0 | 0 | 0 | 0 | 2 | 44.3358 | 40 | NA | NA | threatened | 0.1034 | 0.31 | 45 | 0.548 | 0.1047 | 1 |
| Chigua_bernalii | CR | 1 | 0 | 0 | 1 | 1 | 0 | 0 | 0 | 0 | 3 | 92.77871 | NA | 75 | 150 | threatened | 1 | 1.4 | NA | 0.6 | 1.6 | NA |
| Cycas_aculeata | VU | 1 | 0 | 0 | 0 | 0 | 0 | 0 | 0 | 0 | 1 | 18.227 | 10 | NA | NA | threatened | NA | NA | 30 | 0.15 | 0.18 | 1 |
| Cycas_angulata | LC | NA | NA | NA | NA | NA | NA | NA | NA | NA | 0 | 15.77084 | NA | 0 | 30 | non-threatened | 5 | 12 | NA | 0.15 | 0.25 | 1 |
| Cycas_annaikalensis | CR | 0 | 0 | 0 | 1 | 0 | 0 | 0 | 0 | 0 | 1 | 20.23621 | NA | NA | 940 | threatened | NA | 5 | 40 | 0.19 | 0.61 | 1 |
| Cycas_apoa | NT | 1 | 0 | 0 | 0 | 0 | 0 | 0 | 0 | 0 | 1 | 15.77197 | NA | NA | NA | non-threatened | NA | 2.5 | 40 | NA | NA | 3 |
| Cycas_arenicola | NT | NA | NA | NA | NA | NA | NA | NA | NA | NA | 0 | 16.41264 | NA | NA | NA | non-threatened | 1.5 | 2.5 | 40 | 0.15 | 0.2 | 1 |
| Cycas_armstrongii | VU | 1 | 0 | 0 | 0 | 0 | 0 | 0 | 0 | 1 | 2 | 29.42157 | NA | NA | NA | threatened | 3 | 6 | 40 | 0.05 | 0.11 | 1 |
| Cycas_arnhemica | LC | 0 | 0 | 0 | 0 | 0 | 0 | 0 | 0 | 1 | 1 | 32.74662 | NA | NA | NA | non-threatened | 1.5 | 2.5 | 40 | 0.12 | 0.2 | 1 |
| Cycas_badensis | NT | 1 | 0 | 0 | 0 | 0 | 0 | 0 | 0 | 0 | 1 | 17.56073 | NA | NA | NA | non-threatened | NA | 8 | 40 | NA | NA | 5 |
| Cycas_balansae | NT | 1 | 0 | 0 | 1 | 0 | 0 | 0 | 0 | 0 | 2 | 60.96541 | NA | 100 | 800 | non-threatened | NA | NA | 40 | 0.12 | 0.2 | 1 |
| Cycas_basaltica | LC | 0 | 0 | 0 | 1 | 0 | 0 | 0 | 0 | 0 | 1 | 14.90585 | NA | 230 | 260 | non-threatened | 2 | 4 | 40 | 0.15 | 0.23 | 1 |
| Cycas_beddomei | EN | 1 | 0 | 1 | 0 | 0 | 1 | 0 | 0 | 1 | 4 | 25.56325 | 388 | 300 | 900 | threatened | NA | 2 | 40 | 0.12 | 0.23 | 1 |
| Cycas_bifida | VU | 1 | 0 | 0 | 1 | 0 | 0 | 0 | 0 | 0 | 2 | 18.72094 | NA | 100 | 300 | threatened | NA | NA | 40 | NA | NA | 5 |
| Cycas_bougainvilleana | NT | NA | NA | NA | NA | NA | NA | NA | NA | NA | 0 | 24.69741 | NA | NA | NA | non-threatened | NA | 5 | 40 | NA | NA | 3 |
| Cycas_brachycantha | NT | NA | NA | NA | NA | NA | NA | NA | NA | NA | 0 | 51.24649 | NA | NA | NA | non-threatened | NA | 1 | 40 | 0.09 | 0.12 | 1 |
| Cycas_brunnea | NT | 0 | 0 | 0 | 1 | 0 | 0 | 0 | 0 | 0 | 1 | 30.05926 | NA | NA | NA | non-threatened | 2 | 5 | 40 | 0.17 | 0.23 | 1 |
| Cycas_cairnsiana | VU | 0 | 0 | 0 | 1 | 0 | 0 | 0 | 0 | 0 | 1 | 21.22965 | NA | 450 | 500 | threatened | 2 | 5 | 40 | 0.12 | 0.16 | 1 |
| Cycas_calcicola | LC | 0 | 0 | 0 | 1 | 0 | 0 | 0 | 1 | 1 | 3 | 17.0605 | NA | 123 | 155 | non-threatened | 2 | 5 | 40 | 0.16 | 0.22 | 1 |
| Cycas_campestris | NT | 0 | 0 | 0 | 1 | 0 | 0 | 0 | 0 | 1 | 2 | 19.20191 | 20000 | NA | NA | non-threatened | NA | 2.5 | 40 | NA | 0.2 | 2 |
| Cycas_canalis | LC | 1 | 0 | 0 | 0 | 0 | 1 | 0 | 0 | 1 | 3 | 18.227 | NA | NA | NA | non-threatened | 3 | 5 | 40 | 0.07 | 0.14 | 1 |
| Cycas_candida | EN | 0 | 0 | 0 | 0 | 0 | 0 | 0 | 0 | 1 | 1 | 24.9318 | 55 | NA | NA | threatened | NA | NA | 40 | NA | NA | 1 |
| Cycas_cantafolia | CR | 1 | 0 | 0 | 1 | 0 | 1 | 0 | 0 | 0 | 3 | 17.23081 | NA | NA | NA | threatened | NA | NA | NA | NA | NA | 1 |
| Cycas_chamaoensis | CR | NA | NA | NA | NA | NA | NA | NA | NA | NA | 0 | 14.78945 | NA | NA | NA | threatened | NA | 10 | 40 | 0.14 | 0.28 | 1 |
| Cycas_chamberlainii | EN | 1 | 0 | 0 | 1 | 0 | 0 | 0 | 0 | 0 | 2 | 12.51481 | NA | 615 | 800 | threatened | NA | NA | 40 | NA | NA | 1 |
| Cycas_changjiangensis | EN | 1 | 0 | 0 | 1 | 0 | 0 | 0 | 0 | 0 | 2 | 29.03681 | NA | 600 | 800 | threatened | 0.3 | 2.5 | 40 | NA | 0.2 | 1 |
| Cycas_chevalieri | NT | 0 | 0 | 0 | 1 | 0 | 0 | 0 | 0 | 0 | 1 | 21.68381 | NA | NA | NA | non-threatened | NA | 1.2 | NA | 0.08 | 0.18 | 4 |
| Cycas_circinalis | EN | 1 | 0 | 1 | 1 | 0 | 1 | 0 | 0 | 0 | 4 | 25.56325 | NA | 300 | 1000 | threatened | 6 | 7 | 40 | 0.12 | 0.27 | 5 |
| Cycas_clivicola | LC | 0 | 0 | 0 | 1 | 0 | 0 | 0 | 0 | 0 | 1 | 11.14557 | 300000 | NA | 60 | non-threatened | NA | 8 | 40 | 0.12 | 0.16 | 9 |
| Cycas_collina | VU | 1 | 0 | 0 | 1 | 0 | 0 | 0 | 0 | 0 | 2 | 29.82684 | 20000 | 400 | 900 | threatened | NA | NA | 40 | 0.1 | 0.14 | 1 |
| Cycas_condaoensis | VU | NA | NA | NA | NA | NA | NA | NA | NA | NA | 0 | 26.07318 | 20 | NA | NA | threatened | 0.2 | 2.5 | 40 | 0.14 | 0.17 | 1 |
| Cycas_conferta | NT | 1 | 0 | 0 | 1 | 0 | 0 | 0 | 0 | 0 | 2 | 60.36198 | NA | NA | NA | non-threatened | 4 | 7 | 40 | 0.09 | 0.13 | 1 |
| Cycas_couttsiana | NT | NA | NA | NA | NA | NA | NA | NA | NA | NA | 0 | 31.82362 | NA | NA | 700 | non-threatened | 3 | 7 | 40 | 0.14 | 0.2 | 1 |
| Cycas_cupida | VU | 1 | 0 | 0 | 1 | 0 | 0 | 0 | 0 | 0 | 2 | 20.61617 | 60 | NA | NA | threatened | NA | NA | 40 | NA | NA | 1 |
| Cycas_curranii | CR | 1 | 1 | 0 | 0 | 0 | 0 | 0 | 0 | 0 | 2 | 34.0329 | NA | NA | NA | threatened | NA | 3 | 40 | NA | NA | 1 |
| Cycas_debaoensis | CR | 1 | 1 | 0 | 1 | 0 | 0 | 0 | 0 | 0 | 3 | 10.80662 | NA | 300 | 1300 | threatened | NA | NA | 40 | 0.15 | 0.2 | 1 |
| Cycas_desolata | VU | NA | NA | NA | NA | NA | NA | NA | NA | NA | 0 | 16.41264 | NA | 450 | 550 | threatened | 4 | 7 | 40 | 0.15 | 0.25 | 1 |
| Cycas_diannanensis | VU | NA | NA | NA | NA | NA | NA | NA | NA | NA | 0 | 11.02612 | NA | 600 | 1800 | threatened | NA | 0.03 | 40 | 0.25 | 0.35 | 2 |
| Cycas_dolichophylla | NT | NA | NA | NA | NA | NA | NA | NA | NA | NA | 0 | 18.87451 | NA | NA | NA | non-threatened | NA | 1.5 | 40 | 0.18 | 0.3 | 11 |
| Cycas_edentata | NT | 1 | 0 | 0 | 0 | 0 | 0 | 0 | 0 | 0 | 1 | 13.48673 | 1000 | NA | NA | non-threatened | NA | 10 | 40 | NA | 0.2 | 33 |
| Cycas_elephantipes | EN | 0 | 0 | 0 | 1 | 0 | 0 | 0 | 0 | 0 | 1 | 29.1319 | NA | NA | NA | threatened | 1 | 3 | 40 | 0.15 | 0.2 | 1 |
| Cycas_elongata | EN | 1 | 1 | 0 | 1 | 0 | 0 | 0 | 0 | 0 | 3 | 21.47247 | NA | 50 | 200 | threatened | 2 | 5 | 40 | 0.1 | 0.2 | 5 |
| Cycas_falcata | VU | NA | NA | NA | NA | NA | NA | NA | NA | NA | 0 | 11.02612 | 1350 | NA | NA | threatened | NA | 5 | 40 | 0.12 | 0.3 | 2 |
| Cycas_ferruginea | NT | NA | NA | NA | NA | NA | NA | NA | NA | NA | 0 | 43.33355 | 7215 | NA | NA | non-threatened | NA | 1.2 | 40 | 0.12 | 0.18 | 3 |
| Cycas_fugax | CR | 1 | 0 | 0 | 0 | 0 | 0 | 0 | 0 | 0 | 1 | 37.99666 | NA | NA | 200 | threatened | NA | NA | 40 | 0.08 | 0.12 | 1 |
| Cycas_guizhouensis | VU | 1 | 0 | 1 | 1 | 0 | 0 | 0 | 0 | 0 | 3 | 10.80662 | NA | 400 | 1300 | threatened | NA | 1 | 40 | 0.1 | 0.15 | 3 |
| Cycas_hainanensis | EN | 1 | 1 | 0 | 0 | 0 | 0 | 0 | 0 | 0 | 2 | 34.0329 | NA | 0 | 1200 | threatened | 0.3 | 3.5 | 40 | NA | 0.3 | 1 |
| Cycas_hoabinhensis | EN | 0 | 0 | 0 | 1 | 0 | 0 | 0 | 0 | 0 | 1 | 24.43046 | NA | 50 | 150 | threatened | NA | 0.6 | 40 | 0.05 | 0.08 | 4 |
| Cycas_hongheensis | CR | 0 | 0 | 0 | 1 | 0 | 1 | 0 | 0 | 0 | 2 | 28.09396 | NA | 400 | 600 | threatened | 1 | 3 | 40 | 0.12 | 0.15 | 1 |
| Cycas_inermis | VU | 1 | 0 | 0 | 1 | 0 | 0 | 0 | 0 | 0 | 2 | 37.99666 | NA | NA | NA | threatened | 1.5 | 4 | 40 | 0.08 | 0.14 | 4 |
| Cycas_javana | EN | 1 | 0 | 0 | 0 | 0 | 0 | 0 | 0 | 0 | 1 | 28.4363 | NA | NA | NA | threatened | 2 | 4 | 40 | 0.15 | 0.2 | 3 |
| Cycas_lacrimans | EN | NA | NA | NA | NA | NA | NA | NA | NA | NA | 0 | 14.358 | NA | NA | NA | threatened | 1 | 2 | NA | NA | NA | 1 |
| Cycas_lane-poolei | LC | NA | NA | NA | NA | NA | NA | NA | NA | NA | 0 | 14.37567 | NA | 300 | 370 | non-threatened | 5 | 8 | 40 | 0.12 | 0.15 | 1 |
| Cycas_lindstromii | EN | 1 | 0 | 0 | 1 | 0 | 0 | 0 | 0 | 0 | 2 | 20.23621 | 4280 | 0 | 30 | threatened | NA | NA | 40 | 0.05 | 0.08 | 4 |
| Cycas_litoralis | NT | 1 | 0 | 0 | 0 | 0 | 0 | 0 | 0 | 0 | 1 | 12.58177 | 1000 | NA | NA | non-threatened | NA | NA | 40 | NA | NA | NA |
| Cycas_maconochiei | LC | 0 | 0 | 0 | 0 | 0 | 0 | 0 | 1 | 1 | 2 | 15.93741 | NA | 0 | 40 | non-threatened | 3 | 7 | 40 | 0.09 | 0.15 | 1 |
| Cycas_macrocarpa | VU | 1 | 0 | 0 | 0 | 0 | 0 | 0 | 0 | 0 | 1 | 45.82549 | NA | NA | NA | threatened | NA | 12 | 40 | NA | NA | 6 |
| Cycas_media_ensata | LC | 1 | 0 | 0 | 0 | 0 | 0 | 1 | 0 | 0 | 2 | 15.78573 | NA | 0 | 860 | non-threatened | 3 | 6 | 40 | 0.1 | 0.18 | 1 |
| Cycas_media_media | LC | 1 | 0 | 0 | 0 | 0 | 0 | 1 | 0 | 0 | 2 | 17.49625 | NA | 0 | 860 | non-threatened | 3 | 6 | 40 | 0.1 | 0.18 | NA |
| Cycas_megacarpa | VU | 1 | 0 | 0 | 0 | 0 | 0 | 0 | 0 | 0 | 1 | 18.53765 | NA | 150 | 300 | threatened | 3 | 6 | 40 | 0.08 | 0.14 | 1 |
| Cycas_micholitzii | VU | 1 | 0 | 0 | 0 | 0 | 0 | 0 | 0 | 0 | 1 | 36.43868 | NA | 130 | 600 | threatened | NA | NA | 40 | 0.1 | 0.15 | 3 |
| Cycas_micronesica | EN | 1 | 0 | 0 | 0 | 0 | 0 | 1 | 1 | 0 | 3 | 10.58724 | 11250000 | NA | NA | threatened | 8 | 12 | 40 | 0.14 | 0.25 | 1 |
| Cycas_miquellii | LC | 1 | 0 | 0 | 0 | 0 | 1 | 0 | 0 | 0 | 2 | 21.02826 | NA | 0 | 300 | non-threatened | NA | NA | NA | NA | NA | NA |
| Cycas_montana | NT | 1 | 0 | 0 | 1 | 0 | 0 | 0 | 1 | 0 | 3 | 22.60174 | NA | NA | NA | non-threatened | NA | 1.5 | NA | 0.3 | 0.35 | 1 |
| Cycas_multipinnata | EN | 1 | 0 | 0 | 1 | 0 | 0 | 0 | 0 | 0 | 2 | 18.43632 | 27040 | 200 | 1300 | threatened | NA | NA | 40 | 0.14 | 0.25 | 2 |
| Cycas_nathorstii | VU | 1 | 0 | 1 | 0 | 0 | 0 | 0 | 0 | 0 | 2 | 14.358 | NA | 30 | 300 | threatened | NA | 4.5 | 40 | 0.11 | 0.2 | 2 |
| Cycas_nitida | NT | 1 | 0 | 0 | 0 | 0 | 0 | 0 | 0 | 0 | 1 | 25.68127 | NA | NA | NA | non-threatened | NA | NA | NA | NA | NA | 1 |
| Cycas_nongnoochiae | VU | 0 | 0 | 0 | 1 | 0 | 0 | 0 | 0 | 1 | 2 | 19.79711 | NA | 50 | 100 | threatened | NA | 5 | NA | 0.1 | 0.15 | 1 |
| Cycas_ophiolitica | VU | 1 | 0 | 0 | 1 | 0 | 0 | 1 | 0 | 0 | 3 | 14.90585 | NA | 150 | 250 | threatened | 2 | 7 | 40 | 0.14 | 0.2 | 1 |
| Cycas_orientis | LC | 0 | 0 | 0 | 0 | 0 | 0 | 0 | 0 | 1 | 1 | 28.09396 | NA | NA | NA | non-threatened | 4 | 7 | 40 | 0.08 | 0.14 | 1 |
| Cycas_pachypoda | CR | 0 | 1 | 0 | 1 | 0 | 0 | 0 | 0 | 0 | 2 | 19.21076 | NA | NA | NA | threatened | 0.5 | 1.5 | 40 | 0.12 | 0.17 | 2 |
| Cycas_panzhihuaensis | VU | 1 | 0 | 1 | 1 | 0 | 0 | 0 | 0 | 0 | 3 | 30.05926 | 14500 | 1100 | 2000 | threatened | 1 | 3 | 40 | 0.15 | 0.2 | 2 |
| Cycas_papuana | NT | 0 | 0 | 0 | 0 | 0 | 0 | 0 | 0 | 1 | 1 | 21.47898 | NA | NA | NA | non-threatened | NA | 2.8 | 40 | NA | NA | 2 |
| Cycas_pectinata_A | VU | 0 | 0 | 1 | 0 | 0 | 0 | 0 | 0 | 0 | 1 | 13.66021 | NA | 600 | 1300 | threatened | 1 | 12 | 40 | 0.14 | 0.2 | 17 |
| Cycas_pectinata_B | VU | 0 | 0 | 1 | 0 | 0 | 0 | 0 | 0 | 0 | 1 | 17.56073 | NA | 600 | 1300 | threatened | 1 | 12 | 40 | 0.14 | 0.2 | 17 |
| Cycas_petraea | NT | NA | NA | NA | NA | NA | NA | NA | NA | NA | 0 | 24.43046 | 60 | NA | NA | non-threatened | NA | 6 | 40 | 0.15 | 0.2 | 1 |
| Cycas_platyphylla | EN | 0 | 0 | 0 | 1 | 0 | 0 | 0 | 0 | 0 | 1 | 18.53765 | NA | 400 | 750 | threatened | 2 | 4 | 40 | 0.1 | 0.15 | 1 |
| Cycas_pranburiensis | VU | 0 | 0 | 0 | 1 | 0 | 0 | 0 | 0 | 0 | 1 | 43.33355 | NA | 5 | 30 | threatened | 1 | 3 | 40 | 0.08 | 0.1 | 1 |
| Cycas_pruinosa | LC | NA | NA | NA | NA | NA | NA | NA | NA | NA | 0 | 29.1319 | NA | NA | NA | non-threatened | 1.5 | 2.5 | 40 | 0.15 | 0.35 | 1 |
| Cycas_revoluta | LC | 1 | 0 | 0 | 0 | 0 | 1 | 0 | 0 | 0 | 2 | 23.2936 | NA | 0 | 300 | non-threatened | 0.5 | 2 | 40 | NA | 0.2 | 1 |
| Cycas_riuminiana | EN | 1 | 0 | 0 | 0 | 0 | 0 | 0 | 0 | 0 | 1 | 14.32454 | NA | 615 | 800 | threatened | NA | NA | 40 | NA | NA | 1 |
| Cycas_rumphii | NT | 1 | 0 | 0 | 0 | 0 | 0 | 0 | 0 | 0 | 1 | 14.37567 | NA | 10 | 200 | non-threatened | 3 | 10 | 40 | 0.11 | 0.2 | 11 |
| Cycas_saxatilis | VU | 1 | 0 | 0 | 0 | 0 | 0 | 0 | 0 | 0 | 1 | 19.20191 | NA | NA | NA | threatened | 0.5 | 4 | 40 | NA | NA | 1 |
| Cycas_schumanniana | NT | 1 | 0 | 0 | 0 | 0 | 0 | 0 | 0 | 1 | 2 | 12.8756 | NA | NA | 1600 | non-threatened | NA | 2 | 40 | 0.15 | 0.2 | 4 |
| Cycas_scratchleyana | NT | NA | NA | NA | NA | NA | NA | NA | NA | NA | 0 | 14.13015 | NA | 5 | 900 | non-threatened | 4 | 7 | 40 | 0.12 | 0.2 | 7 |
| Cycas_seemanii | VU | 1 | 0 | 0 | 0 | 0 | 0 | 0 | 0 | 0 | 1 | 21.69839 | NA | 0 | 600 | threatened | 4 | 10 | 40 | 0.1 | 0.2 | 5 |
| Cycas_segmentifida | VU | 0 | 1 | 0 | 0 | 0 | 0 | 0 | 0 | 0 | 1 | 36.43868 | NA | 600 | 900 | threatened | NA | 0.5 | 40 | 0.1 | 0.23 | 4 |
| Cycas_semota | NT | 0 | 0 | 0 | 1 | 0 | 0 | 0 | 0 | 0 | 1 | 30.74311 | NA | NA | NA | non-threatened | NA | 5 | 40 | NA | NA | 1 |
| Cycas_sexseminifera | VU | 0 | 0 | 0 | 1 | 0 | 0 | 0 | 0 | 0 | 1 | 14.11838 | NA | NA | NA | threatened | NA | 0.6 | 40 | 0.06 | 0.15 | 4 |
| Cycas_shanyaensis | VU | NA | NA | NA | NA | NA | NA | NA | NA | NA | 0 | 12.51481 | 10 | 700 | 800 | threatened | 2.1 | 3.1 | 40 | 0.2 | 0.25 | 1 |
| Cycas_siamensis | VU | 1 | 0 | 0 | 1 | 0 | 0 | 0 | 0 | 1 | 3 | 11.14557 | NA | NA | 300 | threatened | NA | 1.5 | 40 | 0.14 | 0.2 | 20 |
| Cycas_silvestris | VU | NA | NA | NA | NA | NA | NA | NA | NA | NA | 0 | 20.74771 | NA | NA | NA | threatened | 0.03 | 0.04 | 40 | 0.1 | 0.15 | 1 |
| Cycas_simplicipinna | NT | 1 | 0 | 0 | 0 | 0 | 0 | 0 | 0 | 0 | 1 | 29.82684 | NA | 600 | 1300 | non-threatened | NA | NA | 40 | 0.08 | 0.14 | 5 |
| Cycas_sundaica | LC | 1 | 0 | 0 | 1 | 0 | 0 | 0 | 1 | 0 | 3 | 14.11838 | NA | NA | NA | non-threatened | NA | 0.05 | 40 | 0.2 | 0.35 | 1 |
| Cycas_szechuanensis | CR | 1 | 0 | 0 | 1 | 0 | 0 | 0 | 0 | 0 | 2 | 42.39772 | NA | NA | NA | threatened | NA | 2 | 40 | 0.15 | 0.25 | 2 |
| Cycas_taitungensis | EN | 0 | 0 | 0 | 1 | 0 | 1 | 0 | 0 | 0 | 2 | 25.68127 | 65 | 400 | 900 | threatened | 0.03 | 0.06 | 40 | 0.25 | 0.3 | 1 |
| Cycas_taiwaniana | EN | 1 | 0 | 0 | 1 | 0 | 0 | 0 | 0 | 0 | 2 | 60.36198 | NA | 400 | 1100 | threatened | NA | 3.5 | 40 | 0.15 | 0.3 | 1 |
| Cycas_tanqingii | NT | 1 | 0 | 0 | 0 | 0 | 0 | 0 | 0 | 0 | 1 | 20.61617 | 80 | NA | 800 | non-threatened | NA | 2 | 40 | 0.25 | 0.3 | 2 |
| Cycas_tansachana | CR | 1 | 0 | 0 | 1 | 0 | 0 | 0 | 0 | 0 | 2 | 15.77084 | 10 | NA | 400 | threatened | 2 | 5 | 40 | 0.1 | 0.18 | 1 |
| Cycas_terryana | VU | 1 | 0 | 0 | 0 | 0 | 0 | 0 | 0 | 0 | 1 | 23.2936 | NA | NA | NA | threatened | NA | NA | 40 | NA | NA | 1 |
| Cycas_thouarsii | LC | 1 | 0 | 0 | 1 | 0 | 0 | 0 | 0 | 0 | 2 | 18.7099 | NA | 0 | 200 | non-threatened | NA | 4 | 40 | NA | 0.1 | 6 |
| Cycas_tropophylla | NT | NA | NA | NA | NA | NA | NA | NA | NA | NA | 0 | 51.24649 | 400 | NA | NA | non-threatened | NA | 1 | 40 | 0.08 | 0.15 | 2 |
| Cycas_tuckeri | VU | 1 | 0 | 0 | 0 | 0 | 0 | 0 | 0 | 1 | 2 | 19.79711 | 15 | NA | NA | threatened | NA | 5 | 40 | NA | NA | 1 |
| Cycas_vespertilio | NT | 0 | 1 | 0 | 0 | 0 | 0 | 0 | 0 | 0 | 1 | 28.4363 | NA | NA | NA | non-threatened | 1 | 3 | 40 | NA | NA | 6 |
| Cycas_wadei | CR | 1 | 0 | 0 | 1 | 0 | 0 | 0 | 0 | 1 | 3 | 19.06555 | NA | 20 | 50 | threatened | NA | 5 | 40 | 0.1 | 0.2 | 1 |
| Cycas_xipholepis | LC | NA | NA | NA | NA | NA | NA | NA | NA | NA | 0 | 15.93741 | NA | NA | NA | non-threatened | NA | 6 | 40 | 0.1 | 0.15 | 1 |
| Cycas_yorkiana | NT | 1 | 0 | 0 | 0 | 0 | 0 | 0 | 0 | 1 | 2 | 15.78573 | 11530 | NA | NA | non-threatened | NA | 4 | 40 | NA | NA | 1 |
| Cycas_zambalensis | CR | 1 | 0 | 1 | 0 | 0 | 0 | 1 | 0 | 1 | 4 | 20.19838 | NA | NA | NA | threatened | NA | 3 | 40 | NA | NA | 1 |
| Cycas_zeylanica | VU | 1 | 0 | 0 | 0 | 0 | 0 | 0 | 0 | 0 | 1 | 10.58724 | NA | 5 | 50 | threatened | 2.3 | 3.1 | 40 | 0.13 | 0.2 | 2 |
| Dioon_angustifolium | VU | NA | NA | NA | NA | NA | NA | NA | NA | NA | 0 | 18.87281 | NA | NA | NA | threatened | NA | NA | 500 | NA | NA | 2 |
| Dioon_argenteum | VU | 1 | 0 | 0 | 0 | 0 | 0 | 0 | 0 | 0 | 1 | 20.82433 | 350 | 1100 | 1600 | threatened | NA | 3 | 500 | 0.18 | 0.32 | 1 |
| Dioon_califanoi | EN | 1 | 0 | 0 | 1 | 0 | 0 | 0 | 0 | 1 | 3 | 24.654 | 126 | NA | NA | threatened | NA | 3 | 500 | NA | 0.3 | 2 |
| Dioon_caputoi | EN | 1 | 0 | 0 | 1 | 0 | 0 | 0 | 0 | 0 | 2 | 60.02895 | NA | NA | NA | threatened | NA | 1 | 500 | NA | 0.25 | 2 |
| Dioon_edule | NT | 1 | 0 | 0 | 1 | 0 | 0 | 0 | 0 | 0 | 2 | 20.61607 | NA | NA | NA | non-threatened | NA | 3 | 700 | NA | 0.3 | 5 |
| Dioon_holmgrenii | EN | 1 | 0 | 0 | 1 | 0 | 0 | 0 | 0 | 0 | 2 | 21.43385 | NA | NA | NA | threatened | NA | 6 | 500 | NA | 0.4 | 1 |
| Dioon_mejiae | LC | 1 | 1 | 0 | 0 | 0 | 0 | 0 | 0 | 0 | 2 | 22.89612 | NA | NA | NA | non-threatened | NA | 1 | 500 | NA | 0.25 | 3 |
| Dioon_merolae | VU | 0 | 0 | 1 | 1 | 0 | 0 | 0 | 1 | 0 | 3 | 22.43078 | NA | NA | NA | threatened | NA | 3 | 500 | 0.25 | 0.4 | 2 |
| Dioon_purpusii | VU | NA | NA | NA | NA | NA | NA | NA | NA | NA | 0 | 21.43385 | NA | 1000 | 1500 | threatened | NA | 5 | 500 | NA | 0.4 | 1 |
| Dioon_rzedowskii | EN | 1 | 0 | 0 | 0 | 0 | 0 | 0 | 0 | 0 | 1 | 20.82433 | 25 | NA | NA | threatened | NA | 5 | 500 | 0.25 | 0.4 | 1 |
| Dioon_sonorense | EN | 0 | 0 | 1 | 1 | 1 | 0 | 0 | 0 | 0 | 3 | 22.43078 | NA | NA | NA | threatened | NA | NA | 500 | NA | NA | 2 |
| Dioon_spinulosum | EN | 1 | 0 | 0 | 1 | 0 | 0 | 0 | 0 | 0 | 2 | 58.81312 | NA | NA | NA | threatened | 5 | 16 | 500 | NA | 0.4 | 2 |
| Dioon_stevensonii | CR | NA | NA | NA | NA | NA | NA | NA | NA | NA | 0 | 20.61607 | NA | NA | NA | threatened | NA | NA | NA | NA | NA | 2 |
| Dioon_tomasellii | VU | 1 | 0 | 0 | 1 | 0 | 0 | 0 | 0 | 0 | 2 | 18.87281 | NA | 600 | 1850 | threatened | NA | 1 | 500 | NA | NA | 3 |
| Encephalartos | |  |  |  |  |  |  |  |  |  |  |  |  |  |  |  |  |  |  |  |  |  |
| chimanimaniensis" | EN | 0 | 0 | 0 | 1 | 0 | 0 | 0 | 0 | 0 | 1 | 26.60507 | NA | 600 | 1100 | threatened | NA | 1.8 | 70 | NA | 0.45 | 2 |
| Encephalartos | |  |  |  |  |  |  |  |  |  |  |  |  |  |  |  |  |  |  |  |  |  |
| fridericiguilielmi" | NT | 0 | 0 | 1 | 1 | 0 | 0 | 0 | 0 | 0 | 2 | 21.40559 | NA | 0 | 600 | threatened | NA | 4 | 70 | 0.4 | 0.6 | 2 |
| Encephalartos_aemulans | CR | 1 | 0 | 0 | 1 | 0 | 0 | 0 | 0 | 0 | 2 | 19.12329 | 295 | 100 | 600 | threatened | NA | 3 | 70 | NA | 0.35 | 1 |
| Encephalartos_altensteinii | VU | 1 | 0 | 1 | 1 | 0 | 0 | 0 | 0 | 0 | 3 | 12.85207 | 450 | 100 | 200 | threatened | 4 | 7 | 70 | 0.25 | 0.35 | 1 |
| Encephalartos_aplanatus | VU | 0 | 0 | 0 | 1 | 0 | 1 | 0 | 0 | 0 | 2 | 21.02188 | NA | 400 | 1400 | threatened | NA | NA | 35 | NA | NA | 1 |
| Encephalartos_arenarius | EN | 1 | 0 | 0 | 1 | 0 | 0 | 0 | 0 | 0 | 2 | 23.34009 | 140 | 1300 | 1500 | EW | NA | 1 | 70 | 0.2 | 0.3 | 1 |
| Encephalartos_barteri | VU | 0 | 0 | 0 | 1 | 1 | 0 | 0 | 0 | 1 | 3 | 15.62878 | NA | 1300 | 2150 | non-threatened | 0.3 | 2.6 | 70 | 0.25 | 0.6 | 3 |
| Encephalartos_brevifoliolatus | EW | 0 | 0 | 0 | 1 | 0 | 0 | 0 | 0 | 0 | 1 | 53.41136 | NA | 300 | 700 | non-threatened | NA | 2.5 | 70 | 0.25 | 0.3 | 1 |
| Encephalartos_bubalinus | NT | 0 | 0 | 0 | 0 | 0 | 1 | 0 | 0 | 0 | 1 | 22.75749 | NA | 500 | 900 | threatened | NA | 2 | 70 | NA | 0.45 | 2 |
| Encephalartos_caffer | NT | 1 | 0 | 0 | 1 | 0 | 0 | 0 | 0 | 0 | 2 | 14.30221 | NA | NA | 1000 | threatened | 0.3 | 0.4 | 35 | 0.2 | 0.25 | 1 |
| Encephalartos_cerinus | CR | 0 | 0 | 0 | 1 | 0 | 1 | 0 | 0 | 0 | 2 | 17.17823 | NA | 800 | 900 | threatened | NA | 0.3 | 35 | NA | 0.25 | 1 |
| Encephalartos_concinnus | EN | 0 | 0 | 1 | 1 | 0 | 0 | 0 | 0 | 0 | 2 | 34.9623 | 58 | 700 | 1500 | threatened | NA | 3 | 70 | NA | 0.45 | 2 |
| Encephalartos_cupidus | CR | 0 | 0 | 0 | 1 | 1 | 1 | 0 | 0 | 1 | 4 | 47.18981 | 290 | 1200 | 1800 | non-threatened | NA | 0.75 | 200 | 0.2 | 0.3 | 1 |
| Encephalartos_cycadifolius | LC | 0 | 0 | 0 | 0 | 0 | 1 | 0 | 0 | 1 | 2 | 13.48624 | NA | 1200 | 1950 | threatened | 0.5 | 1.5 | 500 | NA | 0.25 | 1 |
| Encephalartos_delucanus | EN | 1 | 0 | 0 | 1 | 0 | 0 | 0 | 0 | 1 | 3 | 30.92973 | NA | 1100 | 1500 | threatened | NA | 0.12 | 70 | 0.1 | 0.2 | 1 |
| Encephalartos_dolomiticus | CR | 0 | 0 | 0 | 1 | 0 | 1 | 0 | 0 | 0 | 2 | 47.18981 | 0.3 | NA | 700 | threatened | NA | 2 | 70 | NA | 0.4 | 1 |
| Encephalartos_dyerianus | CR | 0 | 0 | 0 | 1 | 0 | 1 | 0 | 0 | 0 | 2 | 12.85207 | 5 | NA | 1000 | threatened | NA | 4 | 70 | NA | 0.6 | 1 |
| Encephalartos_equatorialis | CR | 0 | 0 | 0 | 1 | 0 | 1 | 0 | 0 | 1 | 3 | 28.26662 | NA | 1400 | 1500 | threatened | NA | 6 | 70 | 0.4 | 0.6 | 1 |
| Encephalartos_eugenemaraisii | EN | 0 | 0 | 0 | 1 | 0 | 1 | 0 | 0 | 0 | 2 | 35.18108 | NA | 20 | 100 | non-threatened | 2.5 | 4 | 70 | 0.3 | 0.45 | 1 |
| Encephalartos_ferox | NT | 1 | 0 | 0 | 1 | 0 | 0 | 0 | 0 | 0 | 2 | 19.8147 | NA | 700 | 1400 | non-threatened | 1 | 2 | 70 | NA | 0.3 | 2 |
| Encephalartos_ghellincki | VU | 0 | 0 | 0 | 1 | 0 | 0 | 0 | 0 | 1 | 2 | 19.8147 | NA | 700 | 2400 | threatened | NA | 3 | 70 | 0.3 | 0.4 | 2 |
| Encephalartos_gratus | VU | 1 | 0 | 0 | 0 | 0 | 0 | 0 | 0 | 0 | 1 | 52.39442 | NA | 650 | 900 | threatened | NA | 2.5 | 70 | NA | 0.6 | 2 |
| Encephalartos_heenanii | CR | 1 | 0 | 0 | 1 | 0 | 1 | 0 | 0 | 1 | 4 | 17.77268 | 300 | 750 | 1750 | threatened | 2 | 4 | 70 | 0.25 | 0.35 | 2 |
| Encephalartos_hildebrandtii | NT | 1 | 0 | 1 | 0 | 0 | 1 | 0 | 0 | 0 | 3 | 16.6309 | NA | 0 | 600 | non-threatened | NA | 6 | 70 | NA | 0.6 | 5 |
| Encephalartos_hirsutus | CR | 0 | 0 | 0 | 1 | 0 | 0 | 0 | 0 | 0 | 1 | 23.22916 | NA | 800 | 1000 | threatened | NA | 4 | 70 | 0.35 | 0.4 | 1 |
| Encephalartos_horridus | EN | 1 | 0 | 0 | 1 | 0 | 0 | 0 | 0 | 0 | 2 | 13.90459 | NA | 100 | 400 | threatened | NA | 0.3 | 200 | 0.2 | 0.3 | 1 |
| Encephalartos_humilis | VU | 1 | 0 | 0 | 0 | 0 | 0 | 0 | 0 | 1 | 2 | 24.40141 | NA | NA | NA | threatened | 0.35 | 0.5 | NA | 0.13 | 0.18 | 1 |
| Encephalartos_inopinus | CR | 0 | 0 | 0 | 1 | 0 | 1 | 0 | 0 | 0 | 2 | 46.26647 | NA | 600 | 800 | threatened | 2 | 3 | 200 | 0.17 | 0.25 | 1 |
| Encephalartos_ituriensis | NT | 0 | 0 | 0 | 1 | 0 | 0 | 0 | 0 | 0 | 1 | 27.00078 | NA | 1100 | 1200 | non-threatened | NA | 6 | 70 | NA | 0.5 | 2 |
| Encephalartos_kisambo | EN | 1 | 0 | 1 | 1 | 0 | 0 | 0 | 0 | 0 | 3 | 11.67276 | NA | 800 | 1800 | threatened | NA | 4 | 70 | NA | 0.6 | 1 |
| Encephalartos_laevifolius | CR | 1 | 1 | 1 | 1 | 0 | 0 | 0 | 0 | 0 | 4 | 24.40141 | NA | 950 | 1800 | threatened | 3 | 4 | 70 | 0.25 | 0.35 | 5 |
| Encephalartos_lanatus | NT | 1 | 1 | 0 | 0 | 0 | 0 | 0 | 0 | 0 | 2 | 34.20526 | NA | 1200 | 1500 | non-threatened | 1 | 2 | 70 | 0.25 | 0.3 | 1 |
| Encephalartos_latifrons | CR | 1 | 0 | 0 | 1 | 0 | 1 | 0 | 0 | 0 | 3 | 22.23855 | NA | 200 | 600 | threatened | 0.025 | 3 | 100 | NA | NA | 1 |
| Encephalartos_laurentianus | NT | 0 | 0 | 0 | 1 | 0 | 0 | 0 | 0 | 0 | 1 | 17.44729 | NA | 450 | 550 | non-threatened | NA | 15 | 70 | NA | 1 | 2 |
| Encephalartos_lebomboensis | EN | 0 | 1 | 1 | 1 | 0 | 0 | 0 | 0 | 0 | 3 | 20.10179 | NA | 500 | 1000 | threatened | 3 | 5 | 70 | NA | 0.3 | 3 |
| Encephalartos_lehmannii | NT | 0 | 0 | 0 | 1 | 1 | 0 | 1 | 0 | 0 | 3 | 21.01598 | NA | 400 | 1000 | non-threatened | 1.5 | 3 | 200 | 0.25 | 0.45 | 1 |
| Encephalartos_longifolius | NT | 1 | 0 | 0 | 0 | 0 | 0 | 0 | 0 | 0 | 1 | 30.9371 | NA | 200 | 700 | non-threatened | 3 | 4 | 70 | 0.3 | 0.4 | 1 |
| Encephalartos_mackenziei | NT | 0 | 0 | 0 | 1 | 0 | 0 | 0 | 0 | 0 | 1 | 24.78282 | NA | 1800 | 2000 | non-threatened | 1.5 | 2.5 | 70 | NA | NA | 1 |
| Encephalartos_macrostrobilus | EN | 0 | 1 | 0 | 0 | 0 | 0 | 1 | 0 | 0 | 2 | 13.7809 | 50 | 900 | 1400 | threatened | NA | 2.5 | 70 | 0.3 | 0.4 | 1 |
| Encephalartos_manikensis | VU | 0 | 0 | 0 | 1 | 0 | 0 | 0 | 0 | 0 | 1 | 16.90228 | NA | 600 | 1400 | threatened | NA | 1.5 | 70 | NA | 0.3 | 2 |
| Encephalartos_marunguensis | VU | 0 | 0 | 0 | 1 | 0 | 0 | 0 | 0 | 1 | 2 | 27.00078 | 7500 | 1400 | 1700 | threatened | NA | 0.4 | 70 | NA | 0.15 | 1 |
| Encephalartos_middleburgensis | CR | 1 | 0 | 0 | 1 | 0 | 1 | 0 | 0 | 1 | 4 | 34.9623 | NA | 1100 | 1400 | threatened | NA | 7 | 70 | 0.3 | 0.45 | 1 |
| Encephalartos_msinganus | CR | 1 | 0 | 0 | 1 | 0 | 1 | 0 | 0 | 0 | 3 | 17.77268 | 10 | 900 | 1200 | threatened | NA | 3 | 70 | NA | 0.35 | 1 |
| Encephalartos_munchii | CR | 0 | 0 | 0 | 1 | 0 | 1 | 0 | 0 | 0 | 2 | 13.48624 | 3 | 1000 | 1100 | threatened | NA | 1 | 70 | NA | 0.35 | 1 |
| Encephalartos_natalensis | NT | 0 | 0 | 1 | 1 | 0 | 0 | 0 | 0 | 0 | 2 | 21.40559 | NA | 200 | 1200 | non-threatened | 3 | 6.5 | 70 | 0.25 | 0.4 | 1 |
| Encephalartos_ngoyanus | VU | 0 | 0 | 0 | 1 | 0 | 0 | 1 | 0 | 0 | 2 | 53.41136 | NA | 200 | 600 | threatened | NA | 0.3 | 35 | NA | 0.2 | 2 |
| Encephalartos_nubimontanus | NA | NA | NA | NA | NA | NA | NA | NA | NA | NA | 0 | 34.26229 | NA | NA | 1000 | EW | NA | 2.5 | NA | 0.35 | 0.4 | 1 |
| Encephalartos_paucidentatis | VU | 1 | 0 | 0 | 1 | 0 | 0 | 0 | 0 | 0 | 2 | 23.34009 | 424 | 1000 | 1500 | threatened | 6 | 7 | 70 | 0.4 | 0.7 | 2 |
| Encephalartos_poggei | LC | 0 | 0 | 1 | 0 | 0 | 0 | 0 | 0 | 0 | 1 | 30.92973 | NA | 500 | 1000 | non-threatened | NA | 2 | 70 | NA | 0.3 | 3 |
| Encephalartos_princeps | VU | 1 | 0 | 0 | 1 | 0 | 0 | 0 | 0 | 0 | 2 | 22.75749 | 1870 | 200 | 800 | threatened | 3 | 5 | 70 | 0.3 | 0.4 | 1 |
| Encephalartos_pterogononus | CR | 0 | 0 | 0 | 1 | 0 | 1 | 0 | 0 | 0 | 2 | 24.90164 | 35 | 700 | 1000 | threatened | NA | 1.5 | 70 | NA | 0.4 | 1 |
| Encephalartos_relictus | EW | 0 | 0 | 0 | 1 | 0 | 0 | 0 | 0 | 0 | 1 | 12.56742 | NA | 400 | 600 | EW | NA | NA | NA | NA | NA | 1 |
| Encephalartos_schaijesii | VU | 0 | 0 | 0 | 0 | 0 | 1 | 0 | 0 | 1 | 2 | 12.56742 | NA | 1450 | 1500 | threatened | 0.11 | 0.25 | 500 | 0.2 | 0.33 | 1 |
| Encephalartos_schmitzii | VU | 1 | 0 | 0 | 1 | 0 | 0 | 0 | 0 | 1 | 3 | 19.82233 | NA | 1000 | 1400 | threatened | NA | 0.3 | 500 | NA | 0.2 | 2 |
| Encephalartos_sclavoi | CR | 1 | 0 | 1 | 1 | 0 | 1 | 0 | 0 | 1 | 5 | 11.67276 | NA | 1800 | 2100 | threatened | NA | 1 | 70 | NA | 0.35 | 1 |
| Encephalartos_senticosus | VU | 0 | 0 | 0 | 1 | 0 | 0 | 0 | 0 | 0 | 1 | 20.10179 | NA | 300 | 800 | threatened | NA | 4 | 70 | NA | 0.3 | 2 |
| Encephalartos_septentrionalis | NT | 1 | 0 | 0 | 1 | 1 | 0 | 0 | 0 | 0 | 3 | 13.7809 | NA | 500 | 2500 | non-threatened | NA | 2.5 | 70 | NA | 0.75 | 2 |
| Encephalartos_tegulaneus | LC | 0 | 0 | 1 | 1 | 0 | 0 | 0 | 0 | 0 | 2 | 13.12263 | NA | 1400 | 2300 | non-threatened | NA | 10 | 70 | NA | 0.6 | 1 |
| Encephalartos_transvenosus | LC | 1 | 0 | 0 | 1 | 0 | 0 | 0 | 0 | 0 | 2 | 23.22916 | NA | 600 | 1500 | non-threatened | 5 | 13 | 70 | 0.4 | 0.5 | 1 |
| Encephalartos_trispinosus | VU | 0 | 0 | 0 | 1 | 0 | 0 | 0 | 0 | 0 | 1 | 13.90459 | NA | 100 | 600 | threatened | NA | 1 | NA | 0.25 | 0.3 | 1 |
| Encephalartos_turneri | LC | NA | NA | NA | NA | NA | NA | NA | NA | NA | 0 | 45.29299 | NA | 600 | 1200 | non-threatened | NA | 3 | 70 | NA | 0.8 | 1 |
| Encephalartos_umbeluziensis | EN | 0 | 0 | 0 | 1 | 0 | 0 | 0 | 0 | 0 | 1 | 28.26662 | 336 | 50 | 120 | threatened | NA | 0.3 | 35 | 0.2 | 0.25 | 2 |
| Encephalartos_villosus | LC | 1 | 0 | 0 | 1 | 0 | 0 | 0 | 0 | 0 | 2 | 19.12329 | NA | 100 | 600 | non-threatened | NA | 0.3 | 35 | NA | 0.2 | 3 |
| Encephalartos_whitelockii | CR | 1 | 0 | 0 | 1 | 0 | 0 | 1 | 0 | 0 | 3 | 34.44208 | NA | 1000 | 1300 | threatened | NA | 4 | 70 | 0.35 | 0.4 | 1 |
| Encephalartos_woodii | EW | 1 | 0 | 0 | 0 | 0 | 0 | 0 | 0 | 0 | 1 | 30.9371 | NA | NA | NA | EW | 3 | 6 | NA | 0.4 | 0.6 | 1 |
| Lepidozamia_hopei | LC | NA | NA | NA | NA | NA | NA | NA | NA | NA | 0 | 43.35002 | NA | 0 | 1000 | non-threatened | NA | 17 | 100 | NA | 0.5 | 1 |
| Lepidozamia_peroffskyana | LC | 1 | 0 | 0 | 0 | 0 | 0 | 0 | 0 | 0 | 1 | 43.35002 | NA | 0 | 1000 | non-threatened | 4 | 7 | 70 | NA | 0.8 | 1 |
| Macrozamia_cardiacensis | VU | NA | NA | NA | NA | NA | NA | NA | NA | NA | 0 | 15.18195 | 14 | 500 | 640 | threatened | NA | 0.4 | 60 | 0.2 | 0.4 | 1 |
| Macrozamia_communis | LC | 1 | 0 | 0 | 0 | 0 | 0 | 0 | 0 | 0 | 1 | 15.78572 | NA | 0 | 300 | non-threatened | NA | 1.5 | 60 | 0.4 | 0.9 | 1 |
| Macrozamia_concinna | LC | 0 | 0 | 0 | 0 | 0 | 0 | 0 | 0 | 1 | 1 | 15.18301 | NA | 800 | 1100 | non-threatened | NA | NA | 60 | 0.08 | 0.15 | 1 |
| Macrozamia_conferta | VU | 0 | 0 | 0 | 1 | 0 | 0 | 0 | 0 | 0 | 1 | 32.28496 | 423 | 600 | 750 | threatened | NA | NA | 60 | 0.15 | 0.3 | 1 |
| Macrozamia_cranei | EN | 1 | 0 | 0 | 0 | 0 | 0 | 0 | 0 | 0 | 1 | 54.65748 | NA | 400 | 600 | threatened | NA | NA | 60 | 0.1 | 0.25 | 1 |
| Macrozamia_crassifolia | VU | NA | NA | NA | NA | NA | NA | NA | NA | NA | 0 | 32.28496 | 160 | 340 | 420 | threatened | NA | NA | 60 | 0.1 | 0.2 | 1 |
| Macrozamia_diplomera | LC | NA | NA | NA | NA | NA | NA | NA | NA | NA | 0 | 24.60612 | NA | NA | 500 | non-threatened | NA | NA | 60 | 0.2 | 0.4 | 1 |
| Macrozamia_douglasii | LC | NA | NA | NA | NA | NA | NA | NA | NA | NA | 0 | 26.54739 | NA | 0 | 150 | non-threatened | NA | 0.6 | 60 | 0.4 | 0.7 | 1 |
| Macrozamia_dyeri | LC | 0 | 0 | 0 | 0 | 0 | 0 | 0 | 0 | 1 | 1 | 28.07962 | NA | NA | NA | non-threatened | 0.4 | 3 | 60 | 0.5 | 1.2 | 1 |
| Macrozamia_elegans | EN | 1 | 0 | 0 | 1 | 0 | 0 | 0 | 0 | 0 | 2 | 33.38726 | 112 | 120 | 150 | threatened | NA | NA | 60 | 0.15 | 0.3 | 1 |
| Macrozamia_fawcettii | NT | 0 | 0 | 0 | 1 | 0 | 0 | 0 | 0 | 0 | 1 | 32.3706 | 5500 | 5 | 550 | non-threatened | NA | NA | 60 | 0.1 | 0.2 | 1 |
| Macrozamia_fearnsidei | LC | 1 | 0 | 0 | 0 | 0 | 0 | 0 | 0 | 0 | 1 | 13.06859 | NA | 300 | 600 | non-threatened | NA | NA | 60 | 0.15 | 0.35 | 1 |
| Macrozamia_flexuosa | EN | 1 | 0 | 0 | 1 | 0 | 0 | 0 | 0 | 0 | 2 | 22.74608 | NA | NA | NA | threatened | NA | NA | 60 | 0.08 | 0.2 | 1 |
| Macrozamia_fraseri | LC | NA | NA | NA | NA | NA | NA | NA | NA | NA | 0 | 29.77538 | NA | NA | NA | non-threatened | NA | 3 | 60 | 0.4 | 0.7 | 1 |
| Macrozamia_glaucophylla | LC | NA | NA | NA | NA | NA | NA | NA | NA | NA | 0 | 24.76862 | NA | NA | NA | non-threatened | NA | NA | 60 | 0.2 | 0.4 | 1 |
| Macrozamia_heteromera | LC | 0 | 0 | 0 | 1 | 0 | 0 | 0 | 0 | 0 | 1 | 15.2723 | NA | NA | 200 | non-threatened | NA | NA | 60 | 0.5 | 0.9 | 1 |
| Macrozamia_humilis | VU | 1 | 0 | 0 | 0 | 0 | 0 | 0 | 0 | 0 | 1 | 45.99116 | NA | NA | 600 | threatened | NA | NA | 60 | 0.18 | 0.28 | 1 |
| Macrozamia_johnsonii | LC | 0 | 1 | 0 | 0 | 0 | 0 | 0 | 0 | 0 | 1 | 54.65748 | 222 | NA | NA | non-threatened | 0.3 | 3 | 60 | 0.5 | 0.9 | 1 |
| Macrozamia_lomandroides | EN | 1 | 0 | 0 | 1 | 0 | 0 | 0 | 0 | 0 | 2 | 12.53673 | NA | NA | NA | threatened | NA | NA | 60 | 0.1 | 0.17 | 1 |
| Macrozamia_longispina | NT | NA | NA | NA | NA | NA | NA | NA | NA | NA | 0 | 23.27557 | 50 | 200 | 700 | non-threatened | NA | 0.3 | 60 | 0.2 | 0.3 | 1 |
| Macrozamia_lucida | LC | NA | NA | NA | NA | NA | NA | NA | NA | NA | 0 | 26.54739 | NA | 30 | 600 | non-threatened | NA | NA | 60 | 0.08 | 0.2 | 1 |
| Macrozamia_macdonnelli | LC | 0 | 0 | 0 | 1 | 0 | 1 | 0 | 0 | 0 | 2 | 15.01719 | NA | NA | NA | non-threatened | 0.4 | 3 | 60 | 0.6 | 0.8 | 1 |
| Macrozamia_machinii | VU | 0 | 0 | 0 | 1 | 0 | 0 | 0 | 0 | 0 | 1 | 33.38726 | 460 | 320 | 460 | threatened | NA | NA | 60 | 0.2 | 0.3 | 1 |
| Macrozamia_macleayi | LC | NA | NA | NA | NA | NA | NA | NA | NA | NA | 0 | 12.53673 | NA | 100 | 500 | non-threatened | NA | 0.4 | 60 | 0.3 | 0.4 | 1 |
| Macrozamia_miquelii | LC | NA | NA | NA | NA | NA | NA | NA | NA | NA | 0 | 13.06859 | NA | 0 | 500 | non-threatened | NA | NA | 60 | 0.2 | 0.4 | 1 |
| Macrozamia_montana | LC | NA | NA | NA | NA | NA | NA | NA | NA | NA | 0 | 13.9143 | NA | NA | NA | non-threatened | NA | 0.6 | 60 | 0.25 | 0.45 | 1 |
| Macrozamia_moorei | NT | 1 | 0 | 0 | 0 | 0 | 0 | 0 | 0 | 0 | 1 | 16.16684 | NA | 300 | 500 | non-threatened | 2 | 7 | 60 | 0.5 | 0.8 | 1 |
| Macrozamia_mountperriensis | LC | 1 | 0 | 0 | 0 | 0 | 0 | 0 | 0 | 0 | 1 | 27.25841 | NA | 50 | 400 | non-threatened | NA | NA | 60 | 0.25 | 0.4 | 1 |
| Macrozamia_occidua | VU | 0 | 0 | 0 | 1 | 0 | 0 | 0 | 0 | 0 | 1 | 15.18301 | 10 | 800 | 1000 | threatened | NA | NA | 60 | 0.1 | 0.2 | 1 |
| Macrozamia_parcifolia | VU | 0 | 0 | 0 | 1 | 0 | 1 | 0 | 0 | 1 | 3 | 15.17074 | NA | 60 | 220 | threatened | NA | NA | 60 | 0.1 | 0.2 | 1 |
| Macrozamia_pauliguilielmi | EN | 1 | 0 | 0 | 1 | 0 | 0 | 0 | 0 | 0 | 2 | 18.55799 | NA | 5 | 25 | threatened | NA | NA | 60 | 0.1 | 0.2 | 1 |
| Macrozamia_platyrhachis | VU | 1 | 0 | 0 | 0 | 0 | 1 | 0 | 0 | 0 | 2 | 15.78572 | NA | NA | NA | threatened | NA | NA | 60 | 0.25 | 0.6 | 1 |
| Macrozamia_plurinervia | EN | 1 | 0 | 0 | 1 | 0 | 1 | 0 | 0 | 1 | 4 | 15.7715 | NA | NA | NA | threatened | NA | NA | 60 | 0.2 | 0.3 | 1 |
| Macrozamia_polymorpha | LC | NA | NA | NA | NA | NA | NA | NA | NA | NA | 0 | 18.55799 | NA | NA | NA | non-threatened | NA | NA | 60 | 0.1 | 0.25 | 1 |
| Macrozamia_reducta | LC | NA | NA | NA | NA | NA | NA | NA | NA | NA | 0 | 13.9143 | NA | NA | NA | non-threatened | NA | 0.4 | 60 | 0.2 | 0.4 | 1 |
| Macrozamia_riedlei | LC | NA | NA | NA | NA | NA | NA | NA | NA | NA | 0 | 17.26147 | NA | NA | NA | non-threatened | NA | 0.3 | 60 | 0.25 | 0.4 | 1 |
| Macrozamia_secunda | VU | NA | NA | NA | NA | NA | NA | NA | NA | NA | 0 | 24.76862 | NA | NA | NA | threatened | NA | NA | 60 | 0.08 | 0.15 | 1 |
| Macrozamia_serpentine | NT | NA | NA | NA | NA | NA | NA | NA | NA | NA | 0 | 15.18195 | 850 | NA | NA | non-threatened | NA | NA | 60 | NA | NA | 1 |
| Macrozamia_spiralis | EN | NA | NA | NA | NA | NA | NA | NA | NA | NA | 0 | 25.61488 | NA | NA | NA | threatened | NA | NA | 60 | 0.08 | 0.2 | 1 |
| Macrozamia_stenomera | NT | 1 | 0 | 0 | 1 | 0 | 0 | 0 | 0 | 0 | 2 | 22.74608 | NA | NA | NA | non-threatened | NA | NA | 60 | 0.1 | 0.25 | 1 |
| Macrozamia_viridis | EN | 1 | 0 | 0 | 0 | 0 | 0 | 0 | 0 | 1 | 2 | 15.17074 | 1000 | NA | NA | threatened | NA | NA | 60 | 0.1 | 0.2 | 1 |
| Microcycas_calocoma | CR | 1 | 0 | 1 | 0 | 0 | 1 | 0 | 0 | 0 | 3 | 98.76153 | NA | NA | NA | threatened | NA | 10 | 100 | NA | 0.6 | 1 |
| Stangeria_eriopus | VU | 1 | 1 | 1 | 1 | 0 | 0 | 1 | 0 | 0 | 5 | 86.06692 | NA | 10 | 750 | threatened | NA | NA | 30 | NA | NA | 2 |
| Zamia_acuminata | VU | 1 | 0 | 0 | 0 | 0 | 0 | 0 | 0 | 0 | 1 | 32.43504 | NA | NA | NA | threatened | NA | 0.4 | 30 | 0.07 | 0.08 | 3 |
| Zamia_amazonum | NT | NA | NA | NA | NA | NA | NA | NA | NA | NA | 0 | 18.789 | NA | NA | NA | non-threatened | NA | 2.5 | 30 | 0.03 | 0.08 | 7 |
| Zamia_amblyphyllidia | VU | 1 | 0 | 0 | 1 | 0 | 0 | 0 | 0 | 0 | 2 | 14.68817 | NA | NA | NA | threatened | NA | NA | 30 | NA | 0.2 | NA |
| Zamia_amplifolia | CR | 1 | 0 | 0 | 0 | 0 | 0 | 0 | 0 | 0 | 1 | 21.18936 | NA | NA | NA | threatened | NA | 2.5 | 30 | NA | NA | 1 |
| Zamia_angustifolia | VU | 1 | 0 | 0 | 0 | 0 | 0 | 0 | 0 | 0 | 1 | 28.15189 | 9000 | NA | NA | threatened | NA | NA | 30 | NA | NA | 4 |
| Zamia_boliviana | NT | NA | NA | NA | NA | NA | NA | NA | NA | NA | 0 | 26.80184 | NA | NA | NA | non-threatened | NA | NA | 30 | 0.03 | 0.1 | 2 |
| Zamia_chigua | NT | NA | NA | NA | NA | NA | NA | NA | NA | NA | 0 | 27.525 | NA | NA | NA | non-threatened | NA | 2 | 30 | NA | 0.15 | 2 |
| Zamia_cremnophila | EN | NA | NA | NA | NA | NA | NA | NA | NA | NA | 0 | 16.38093 | 53 | NA | NA | threatened | 10 | 25 | 30 | 0.03 | 0.09 | 1 |
| Zamia_cunaria | VU | 1 | 0 | 0 | 0 | 0 | 0 | 0 | 0 | 0 | 1 | 39.96309 | 3140 | NA | NA | threatened | NA | NA | NA | NA | 0.1 | 4 |
| Zamia_decumbens | CR | 1 | 0 | 0 | 1 | 0 | 0 | 0 | 0 | 0 | 2 | 28.15189 | NA | NA | NA | threatened | NA | 80 | NA | 0.067 | 0.11 | 3 |
| Zamia_disodon | CR | 1 | 0 | 0 | 0 | 0 | 0 | 0 | 0 | 0 | 1 | 16.34563 | NA | NA | NA | threatened | NA | NA | 30 | 0.05 | 0.08 | 1 |
| Zamia_dressleri | EN | 1 | 0 | 0 | 0 | 0 | 0 | 0 | 0 | 0 | 1 | 28.07089 | 2530 | NA | NA | threatened | NA | NA | 30 | 0.03 | 0.05 | 2 |
| Zamia_elegantissima | EN | 1 | 0 | 0 | 0 | 0 | 0 | 0 | 0 | 0 | 1 | 19.60963 | 100 | NA | NA | threatened | NA | NA | 30 | NA | NA | 2 |
| Zamia_encephalartoides | VU | 1 | 0 | 0 | 0 | 0 | 0 | 0 | 0 | 0 | 1 | 25.66209 | 266 | NA | NA | threatened | NA | 2 | 30 | NA | 0.25 | 1 |
| Zamia_fairchildiana | NT | NA | NA | NA | NA | NA | NA | NA | NA | NA | 0 | 24.51522 | NA | NA | NA | non-threatened | 0.5 | 1 | 30 | 0.06 | 0.15 | 3 |
| Zamia_fischeri | EN | 1 | 0 | 0 | 0 | 0 | 0 | 0 | 0 | 0 | 1 | 25.79944 | 2770 | NA | NA | threatened | NA | NA | 30 | 0.02 | 0.08 | 4 |
| Zamia_furfuracea_A | EN | 1 | 0 | 0 | 0 | 0 | 1 | 0 | 0 | 0 | 2 | 33.70254 | 630 | NA | NA | threatened | NA | NA | 30 | NA | 0.2 | 1 |
| Zamia_furfuracea_B | EN | 0 | 0 | 0 | 1 | 0 | 0 | 0 | 0 | 0 | 1 | 21.53129 | 631 | NA | NA | threatened | NA | NA | 30 | NA | 0.2 | 1 |
| Zamia_gentryi | CR | 1 | 0 | 0 | 0 | 0 | 0 | 0 | 0 | 0 | 1 | 26.08393 | 5 | NA | NA | threatened | NA | 1.5 | 30 | 0.05 | 0.15 | 2 |
| Zamia_gomeziana | VU | 1 | 0 | 0 | 0 | 0 | 1 | 0 | 0 | 0 | 2 | 25.3669 | NA | NA | NA | threatened | NA | NA | NA | NA | NA | 1 |
| Zamia_hamannii | CR | 1 | 0 | 1 | 0 | 0 | 0 | 0 | 0 | 0 | 2 | 29.18057 | NA | NA | NA | threatened | NA | 2.4 | NA | 0.075 | 0.2 | 1 |
| Zamia_herrerae | VU | 1 | 0 | 0 | 0 | 0 | 0 | 0 | 0 | 0 | 1 | 52.42318 | NA | NA | NA | threatened | NA | NA | 30 | 0.03 | 0.1 | 6 |
| Zamia_hymenophyllidia | CR | 1 | 0 | 0 | 0 | 0 | 0 | 0 | 0 | 0 | 1 | 34.88073 | NA | NA | NA | threatened | NA | NA | 30 | 0.02 | 0.04 | 2 |
| Zamia_imperialis | CR | 1 | 0 | 0 | 1 | 0 | 0 | 0 | 0 | 0 | 2 | 34.74487 | NA | NA | NA | threatened | NA | 1.1 | NA | NA | 0.22 | 3 |
| Zamia_incognita | VU | 1 | 1 | 0 | 0 | 0 | 0 | 0 | 0 | 0 | 2 | 14.74109 | NA | NA | NA | threatened | NA | NA | NA | NA | NA | 3 |
| Zamia_inermis | CR | 1 | 0 | 0 | 1 | 0 | 1 | 0 | 0 | 1 | 4 | 26.59268 | NA | NA | NA | threatened | 0.7 | 1.5 | 30 | 0.2 | 0.25 | 1 |
| Zamia_integrifolia | NT | 1 | 0 | 0 | 1 | 0 | 0 | 0 | 0 | 0 | 2 | 32.54882 | NA | NA | NA | non-threatened | NA | 1.3 | 30 | NA | 0.06 | 5 |
| Zamia_ipetiensis | EN | 1 | 0 | 0 | 0 | 0 | 0 | 0 | 0 | 0 | 1 | 26.5222 | 50 | NA | NA | threatened | NA | NA | 30 | NA | 0.1 | 2 |
| Zamia_katzeriana | EN | NA | NA | NA | NA | NA | NA | NA | NA | NA | 0 | 34.87335 | NA | NA | NA | threatened | NA | NA | 30 | NA | NA | 3 |
| Zamia_kickxii | CR | NA | NA | NA | NA | NA | NA | NA | NA | NA | 0 | 14.68817 | NA | NA | NA | threatened | NA | NA | 30 | NA | NA | NA |
| Zamia_lacandona | EN | 1 | 0 | 0 | 0 | 0 | 0 | 0 | 0 | 1 | 2 | 38.76277 | 3400 | NA | NA | threatened | 0.15 | 0.6 | 30 | 0.045 | 0.08 | 1 |
| Zamia_lawsoniana | NT | 1 | 0 | 0 | 0 | 0 | 0 | 1 | 0 | 0 | 2 | 33.70254 | NA | NA | NA | non-threatened | NA | NA | 30 | NA | NA | NA |
| Zamia_lecointei | NT | NA | NA | NA | NA | NA | NA | NA | NA | NA | 0 | 22.97657 | NA | NA | NA | non-threatened | NA | NA | 30 | 0.05 | 0.1 | 4 |
| Zamia_lindenii | NT | 1 | 1 | 0 | 0 | 0 | 0 | 0 | 0 | 0 | 2 | 21.50499 | NA | NA | NA | non-threatened | NA | 4 | 30 | 0.1 | 0.3 | 10 |
| Zamia_loddigesii | NT | 1 | 0 | 0 | 0 | 0 | 0 | 1 | 0 | 0 | 2 | 38.73882 | NA | NA | NA | non-threatened | NA | 0.3 | 30 | NA | 0.12 | 6 |
| Zamia_lucayana | EN | 1 | 0 | 0 | 0 | 0 | 0 | 0 | 0 | 0 | 1 | 27.68497 | 13 | NA | NA | threatened | NA | NA | 30 | NA | NA | 2 |
| Zamia_macrochiera | CR | 1 | 0 | 0 | 0 | 0 | 0 | 0 | 0 | 0 | 1 | 45.24072 | NA | NA | NA | threatened | NA | NA | 30 | 0.1 | 0.2 | 1 |
| Zamia_manicata | NT | 1 | 0 | 0 | 0 | 0 | 0 | 0 | 0 | 0 | 1 | 29.18057 | NA | NA | NA | non-threatened | NA | NA | 30 | 0.02 | 0.05 | 3 |
| Zamia_meermanii | EN | 1 | 0 | 0 | 1 | 0 | 0 | 0 | 0 | 1 | 3 | 27.525 | NA | NA | NA | threatened | NA | NA | NA | NA | NA | 2 |
| Zamia_melanorrhachis | EN | 1 | 0 | 0 | 0 | 0 | 0 | 0 | 0 | 0 | 1 | 16.38093 | NA | NA | NA | threatened | NA | NA | 30 | 0.05 | 0.08 | 3 |
| Zamia_montana | CR | 1 | 1 | 0 | 0 | 0 | 0 | 0 | 0 | 0 | 2 | 23.94414 | NA | NA | NA | threatened | 0.5 | 1.5 | 30 | 0.1 | 0.2 | 2 |
| Zamia_monticola | CR | 0 | 1 | 0 | 0 | 0 | 0 | 0 | 0 | 0 | 1 | 31.85847 | NA | NA | NA | threatened | NA | 0.3 | 30 | 0.15 | 0.2 | 1 |
| Zamia_muricata | NT | NA | NA | NA | NA | NA | NA | NA | NA | NA | 0 | 21.18936 | NA | NA | NA | non-threatened | NA | 0.15 | 30 | 0.03 | 0.08 | 8 |
| Zamia_nesophila | CR | 1 | 0 | 0 | 1 | 0 | 0 | 0 | 0 | 0 | 2 | 31.01836 | NA | NA | NA | threatened | NA | 2.8 | NA | 0.06 | 0.24 | 1 |
| Zamia_neurophyllidia | VU | 0 | 0 | 0 | 0 | 1 | 0 | 0 | 0 | 0 | 1 | 48.38625 | NA | NA | NA | threatened | 0.6 | 2 | 30 | 0.05 | 0.12 | 3 |
| Zamia_obliqua | NT | NA | NA | NA | NA | NA | NA | NA | NA | NA | 0 | 19.60963 | NA | NA | NA | non-threatened | 0.5 | 5 | 30 | 0.05 | 0.12 | 4 |
| Zamia_onan-reyesii | CR | 1 | 0 | 0 | 1 | 0 | 0 | 0 | 0 | 0 | 2 | 31.72165 | NA | NA | NA | threatened | NA | NA | NA | NA | NA | 1 |
| Zamia_oreillyi | VU | NA | NA | NA | NA | NA | NA | NA | NA | NA | 0 | 17.15522 | NA | NA | NA | threatened | NA | NA | 30 | NA | NA | 1 |
| Zamia_paucijuga | NT | NA | NA | NA | NA | NA | NA | NA | NA | NA | 0 | 26.08393 | NA | NA | NA | non-threatened | NA | NA | 30 | NA | 0.08 | 6 |
| Zamia_picta | EN | 1 | 0 | 0 | 0 | 0 | 0 | 0 | 0 | 0 | 1 | 25.883 | NA | NA | NA | threatened | NA | NA | 30 | NA | NA | NA |
| Zamia_poeppigiana | NT | NA | NA | NA | NA | NA | NA | NA | NA | NA | 0 | 19.07345 | NA | NA | NA | non-threatened | NA | 3 | 30 | 0.1 | 0.3 | 7 |
| Zamia_portoricensis | EN | 0 | 0 | 0 | 0 | 0 | 1 | 0 | 0 | 0 | 1 | 16.55332 | 220 | NA | NA | threatened | NA | NA | 30 | NA | 0.15 | 1 |
| Zamia_prasina | CR | 1 | 0 | 0 | 0 | 0 | 0 | 0 | 0 | 0 | 1 | 33.45521 | NA | NA | NA | threatened | NA | 0.3 | 30 | NA | 0.1 | 11 |
| Zamia_pseudomonticola | NT | NA | NA | NA | NA | NA | NA | NA | NA | NA | 0 | 21.7473 | NA | NA | NA | non-threatened | NA | 0.3 | 30 | 0.05 | 0.07 | 2 |
| Zamia_pseudoparasitica | NT | 1 | 1 | 0 | 0 | 0 | 0 | 0 | 0 | 0 | 2 | 34.88073 | NA | NA | NA | non-threatened | NA | 1 | 30 | NA | 0.15 | 4 |
| Zamia_pumila | NT | NA | NA | NA | NA | NA | NA | NA | NA | NA | 0 | 25.78335 | NA | NA | NA | non-threatened | NA | NA | 30 | 0.03 | 0.25 | 3 |
| Zamia_purpurea | CR | 1 | 0 | 0 | 0 | 0 | 0 | 0 | 0 | 0 | 1 | 23.94414 | NA | NA | NA | threatened | NA | 0.5 | 30 | NA | 0.04 | 2 |
| Zamia_pygmaea | CR | NA | NA | NA | NA | NA | NA | NA | NA | NA | 0 | 24.13965 | NA | NA | NA | threatened | NA | 0.02 | 30 | NA | 0.04 | 2 |
| Zamia_pyrophylla | CR | 1 | 0 | 0 | 0 | 0 | 0 | 0 | 0 | 0 | 1 | 25.3669 | NA | NA | NA | threatened | NA | NA | NA | NA | 0.1 | 1 |
| Zamia_restrepoi | CR | 1 | 0 | 0 | 1 | 1 | 0 | 0 | 0 | 0 | 3 | 16.34563 | NA | 75 | 150 | threatened | NA | NA | 30 | NA | NA | 1 |
| Zamia_roezlii | NT | 0 | 0 | 1 | 0 | 0 | 0 | 0 | 0 | 0 | 1 | 17.15522 | NA | NA | NA | non-threatened | NA | 7 | 30 | NA | NA | 6 |
| Zamia_sandovalii | NT | NA | NA | NA | NA | NA | NA | NA | NA | NA | 0 | 25.79944 | NA | NA | NA | non-threatened | NA | NA | 30 | NA | NA | 1 |
| Zamia_skinneri | EN | 1 | 0 | 0 | 0 | 0 | 0 | 0 | 0 | 0 | 1 | 47.67762 | 6250 | NA | NA | threatened | 1.2 | 2.4 | 30 | 0.075 | 0.2 | 1 |
| Zamia_soconuscensis | VU | 1 | 0 | 0 | 0 | 0 | 0 | 0 | 0 | 0 | 1 | 21.53129 | NA | NA | NA | threatened | 0.3 | 0.5 | 30 | 0.05 | 0.25 | 1 |
| Zamia_spartea | CR | 1 | 0 | 0 | 0 | 0 | 0 | 0 | 0 | 0 | 1 | 41.82245 | 1235 | NA | NA | threatened | NA | NA | 30 | NA | 0.1 | 1 |
| Zamia_standleyi | VU | NA | NA | NA | NA | NA | NA | NA | NA | NA | 0 | 31.01836 | NA | NA | NA | threatened | NA | NA | 30 | 0.05 | 0.12 | 7 |
| Zamia_stricta | VU | NA | NA | NA | NA | NA | NA | NA | NA | NA | 0 | 16.55332 | 25 | NA | NA | threatened | NA | NA | 30 | NA | NA | 1 |
| Zamia_tolimensis | CR | 1 | 1 | 0 | 0 | 0 | 0 | 0 | 0 | 0 | 2 | 31.59701 | NA | NA | NA | threatened | NA | 4 | NA | 0.1 | 0.3 | 1 |
| Zamia_tuerckheimii | NT | 1 | 0 | 0 | 0 | 0 | 0 | 0 | 0 | 0 | 1 | 27.68497 | NA | NA | NA | non-threatened | 1.5 | 3 | 30 | NA | NA | 1 |
| Zamia_ulei | NT | 0 | 0 | 0 | 1 | 0 | 0 | 0 | 0 | 0 | 1 | 24.13965 | NA | NA | NA | non-threatened | NA | 1 | 30 | 0.04 | 0.06 | 7 |
| Zamia_urep | CR | 1 | 0 | 0 | 0 | 0 | 0 | 0 | 0 | 0 | 1 | 38.73882 | 30 | NA | NA | threatened | 0.4 | 0.5 | 30 | 0.025 | 0.03 | 1 |
| Zamia_variegata | EN | 1 | 0 | 0 | 0 | 0 | 0 | 0 | 0 | 0 | 1 | 36.92315 | NA | NA | NA | threatened | NA | 0.2 | 30 | NA | 0.08 | 4 |
| Zamia_vazquezii | CR | 0 | 1 | 0 | 0 | 0 | 0 | 0 | 0 | 0 | 1 | 66.9213 | NA | NA | NA | threatened | NA | 0.3 | 30 | NA | 0.1 | 1 |
| Zamia_wallisii | CR | 1 | 1 | 0 | 0 | 0 | 1 | 0 | 0 | 0 | 3 | 28.07089 | NA | NA | NA | threatened | NA | NA | 30 | 0.03 | 0.05 | 1 |
| Zamia_lindleyi | DD | 1 | 0 | 0 | 0 | 0 | 0 | 0 | 0 | 0 | 1 | 19.07345 | NA | 1143 | NA | DD | NA | 3 | 30 | 0.1 | 0.3 | 2 |
| Ceratozamia_brevifrons | DD | 1 | 0 | 0 | 0 | 0 | 0 | 1 | 0 | 0 | 2 | 26.68981 | NA | NA | NA | DD | 0.09 | 0.28 | 45 | 0.08 | 0.19 | 1 |
| Cycas_aenigma | DD | 1 | 0 | 0 | 1 | 0 | 0 | 0 | 0 | 0 | 2 | 12.8756 | NA | 1100 | NA | DD | 0.5 | 4 | 40 | NA | NA | 1 |
| Cycas_indicaA. | DD | 0 | 0 | 1 | 0 | 0 | 0 | 0 | 0 | 0 | 1 | 33.75955 | NA | 934 | NA | DD | NA | 4 | 40 | 0.1 | 0.23 | 1 |
| Cycas_sphaerica | DD | 0 | 0 | 1 | 0 | 0 | 0 | 0 | 0 | 0 | 1 | 17.23081 | NA | 300 | 1000 | DD | NA | 5 | 40 | 0.09 | 0.27 | 1 |

**Figure S1** Test for autocorrelation among variables

**Figure S2** Aggregation graphic of the imputed missing data for all threats identified for cycads. Left: Barplots indicating that all threats have the same amount of imputed values; Right: An aggregation plot, showing all existing combinations of imputed (orange) and observed (blue) values. Far Right: small barplot showing the frequencies of different combinations. Threats are coded as follows: Hab_Des = habitat destruction; Def = deforestation; Med = medicinal uses; Ove = over-collection; Fl_Dr = flood/drought; Rep = reproduction failure; Gr = grazing; Inv = invasive species; No_th = number of threats

**Figure S3** Aggregation graphic of the imputed missing data for all predictors of extinction risk included in the study. Left: Barplots indicating geographic range (km^2^) and Minimum height have the largest amount of imputed values; Right: An aggregation plot, showing all existing combinations of imputed (orange) and observed (blue) values. Far Right: small barplot showing the frequencies of different combinations. Overall, the aggregation plot shows that a species for which the geographic range (km2) is missing will also likely lack data on altitude and height. The predictors are: ED = evolutionary distinctiveness; km2 = geographic range measured as surface area; al_mi = minimum altitude; al_ma = maximum altitude; H_min = minimum height; H_max = maximum height; Gen = generation time; D_mi = minimum diameter; D_ma = maximum diameter; Geo = geographic range measured as number of locations of species occurrence.

**Figure S4** Aggregation graphic of the imputed missing data for some predictors of extinction risk. These predictors are those that have minimum and maximum values as indicated in Fig. S3 (e.g. minimum and maximum height); the difference with Fig. S3 is that we only show maximum value of predictors unlike in Fig. S3 where both maximum and minimum values are shown. Left: Barplots indicating geographic range (km^2^) and Minimum height have the largest amount of imputed values; Right: An aggregation plot, showing all existing combinations of imputed (orange) and observed (blue) values. Far Right: small barplot showing the frequencies of different combinations. Overall, the aggregation plot shows that a species for which the geographic range (km2) is missing will also likely lack data on altitude and height. The predictors are: ED = evolutionary distinctiveness; km2 = geographic range measured as surface area; al_mi = minimum altitude; al_ma = maximum altitude; H_min = minimum height; H_max = maximum height; Gen = generation time; D_mi = minimum diameter; D_ma = maximum diameter; Geo = geographic range measured as number of locations of species occurrence.
